# Statistical Tools to Optimize the Recovery of Bioactive Compounds from Marine Byproducts

**DOI:** 10.3390/md22040182

**Published:** 2024-04-18

**Authors:** Zenebe Tadesse Tsegay, Sofia Agriopoulou, Moufida Chaari, Slim Smaoui, Theodoros Varzakas

**Affiliations:** 1 Department of Food Science and Post-Harvest Technology, College of Dryland Agriculture and Natural Resources, Mekelle University, Mekelle P.O. Box 231, Ethiopia; ztlovewith73@gmail.com; 2 Department of Food Science and Technology, University of the Peloponnese, Antikalamos, 24100 Kalamata, Greece; s.agriopoulou@uop.gr; 3 Laboratory of Microbial and Enzymatic Biotechnologies and Biomolecules, Center of Biotechnology of Sfax (CBS), University of Sfax, Road of Sidi Mansour Km 6, P.O. Box 1177, Sfax 3018, Tunisia; moufida.chaari97@gmail.com (M.C.); slim.smaoui@cbs.rnrt.tn (S.S.)

**Keywords:** optimization, extraction, parameters, bioactive molecules, seafoods byproducts, green extraction

## Abstract

Techniques for extracting important bioactive molecules from seafood byproducts, viz., bones, heads, skin, frames, fins, shells, guts, and viscera, are receiving emphasis due to the need for better valorization. Employing green extraction technologies for efficient and quality production of these bioactive molecules is also strictly required. Hence, understanding the extraction process parameters to effectively design an applicable optimization strategy could enable these improvements. In this review, statistical optimization strategies applied for the extraction process parameters of obtaining bioactive molecules from seafood byproducts are focused upon. The type of experimental designs and techniques applied to criticize and validate the effects of independent variables on the extraction output are addressed. Dominant parameters studied were the enzyme/substrate ratio, pH, time, temperature, and power of extraction instruments. The yield of bioactive compounds, including long-chain polyunsaturated fatty acids, amino acids, peptides, enzymes, gelatine, collagen, chitin, vitamins, polyphenolic constituents, carotenoids, etc., were the most studied responses. Efficiency and/or economic and quality considerations and their selected optimization strategies that favor the production of potential bioactive molecules were also reviewed.

## 1. Introduction

Studying the nature of the extraction process factors is critically important for an efficient optimization process and for saving costs and time. There are different optimization strategies of the extraction process used to obtain bioactive compounds from seafood byproducts. These strategies can be grouped into classical (one factor at a time) and multivariate (more than one variable at a time) optimization techniques.

Classical optimization strategies in bioactive compound extraction methods have been carried out by controlling the influence of one factor at a time to predict the experimental response, commonly called univariate or one-variable-at-a-time optimization [1]. This is achieved by keeping other extraction variables at a constant level when one parameter is changed. The major disadvantages of one-variable-at-a-time optimization are the interactive effects between variables cannot be studied and use of larger numbers of experimental works, which makes it less applicable due to the high consumption of time and other resources. Classical optimization (univariate) methods are mostly applied for selecting suitable extraction parameters such as extraction mixture or solvent type for particular bioactive compounds. They cannot have robust experimental conditions since they disregard the possible simultaneous interaction of extraction parameters such as the composition of the extraction solvent, solvent volumes used, solvent type, extraction times, and solid/solvent ratio [2]. Simultaneous interaction effects of extraction parameters should be investigated to obtain the required bioactive molecule. This is due to the critical effects of extraction parameters to achieve optimal conditions such as the effect of temperature on a particular solvent; the effect of temperature on extraction time; the effect of extraction time and type of solvent, etc. Studying the interaction effects of such extraction parameters strongly affects the quality and yield of the final bioactive molecule. Univariate statistical strategies have been dominantly applied for screening the extraction parameters of many bioactive molecules. Then, the suitable screened extraction parameters are optimized using multivariate (more than one variable at a time) optimization strategies for better yield and qualities of the bioactive molecule from seafood byproducts. Optimization strategies such as surface response methodology, mixing modelling, and factorial design enhance the quality and performance of bioactive compound extraction techniques. Response surface methodologies (RSMs) used to determine the maximum and minimum values of the extraction factors employ statistical designs such as central composite design (CCD), Box Behnken design (BBD), Doehlert matrix, three-level factorial design, and mixture design. Applying these methods enables us to study the simultaneous effects of extraction parameters, which saves resources and time and even enhances extraction efficiency [2]. For instance, in a study conducted on the microwave-assisted extraction of bioactive fish oil from the heads and fins of fish, an RSM coupled with CCD was applied to optimize the effects of extraction factors (time, microwave power, and solid–liquid ratio) [3].

Optimization is applicable in determining the maximum or minimum values of extraction variables (power of extraction instrument, solid-to-solvent ratio, temperature, time, the composition of the extraction solvent, etc.), considering quality, yield, and cost of the expected response (output). Many extraction instruments are tested based on optimization techniques to validate their efficiencies, time, and processing cost. During a solid–liquid extraction, the solvent plays a great role in selectivity in which its polarity directly affects the solute to be extracted [4]. Hence, optimizing the type of solvent for selecting the appropriate extracting liquid is very significant. Optimization of the extraction of protein hydrolysates from shrimp (*Metapenaeus dobson*) head waste was carried out using RSMs in order to determine the optimum extraction pH, temperature, and enzyme/substrate ratio for better antioxidant activity [5].

The extraction methods of bioactive compounds from seafood byproducts are broadly applied using traditional (such as wet pressing and extraction using solvents or heat) and green, novel, and sustainable methods (such as enzymatic hydrolysis, microwave-assisted extraction, and supercritical carbon dioxide (SC–CO_2_) extraction techniques). These green and novel methods are more applicable in quality production and for saving extraction energy, resources, time, and reducing associated environmental problems [6]. Moreover, non-thermal extraction methods of bioactive compounds such as membrane technology, pulsed electric field, high hydrostatic pressure, microwave-assisted extraction, cold atmospheric plasma extraction, and dense-phase carbon dioxide are promising to recover extraneous chemical free bioactive compounds [7,8,9]. Other non-thermal extraction techniques employed combined extraction methods for comparison as well as for purification of the required bioactive molecule [10]. Membrane ultrafiltration was applied for the purification of bioactive peptides from codfish blood and sardine cooking wastewater [11]. Membrane sizes and appropriate pressure that achieve larger molecules of protein/peptides were the main factors considered for quality production. Extraction methods using the traditional, green, and novel methods and their controlling parameters are summarized later.

The six principles of green extraction for natural products are the application of selective varieties and use of renewable plant resources, water, or agro-solvents; the use of innovative technologies that optimize energy consumption; utilizing bio- and agro-refining industry to produce co-products; minimizing the number of unit operations for convenient, robust, and controlled processes; and the preservation of extracted bioactive compounds from contamination and biodegradation [12]. Green extraction methods of bioactive compounds are designed to apply non-thermal/modern extraction techniques and use green solvents. This aims to reduce energy consumption, allow the use of new-generation solvents, limit waste (conversion into co-products) to minimize environmental pollution, ensure high quality of the required product, and result in non-hazardous extraction processes. Most of these non-thermal extraction methods and greener extraction procedures demand optimized processes for quality and better future production [8]. Green extraction processes use alternative solvents such as natural and unnatural deep eutectic solvents and ionic liquids as well as organic/non-polar solvents. These green solvents are efficient for the extraction of organic, polymeric bio-compounds and inorganic compounds containing bioactive molecules, which can be applied to food and pharmaceutical formulations [13]. From our review, studies are limited in clearly specifying which of these green solvents are suitable for extracting every bioactive compound from seafood byproducts, except for the application of natural and unnatural deep eutectic solvents such as malonic acid, thiourea, glycerol, and urea for the extraction of chitin from lobster shells and shrimp shells [14].

Efficiency and/or economic and quality considerations are other important issues emphasized by researchers when choosing statistical optimization methods. The best optimization strategies enable efficient exploration of eco-friendly and cost-effective extraction methods. These also maximize the recovery of valuable bioactive compounds. Optimization strategies used to optimize unconventional and/or green solvent extraction methods are the most appropriate for extracting bioactive substances [15]. RSMs have been dominantly applied to optimize the utilization of processing materials, extraction time, and proper solvents. In particular, BBD coupled with an RSM was reported as efficient and economical to optimize the enzymatic hydrolysis variables. This strategy maximized the degree of deproteinization of carotenoprotein production from shrimp head waste and shrimp shell waste. This carotenoprotein production has shown attractive amino acid composition, color, and functional properties [16]. Moreover, an RSM was employed to optimize the extraction method applied to supercritical extraction combined with co-solvents for better astaxanthin yield and total carotenoid content. This optimization strategy efficiently recovered astaxanthin and lipids from Atlantic shrimp byproducts (*Pandalus borealis*) [17].

This review aims to provide an overview of statistical optimization strategies applied for the extraction process parameters of obtaining bioactive molecules from seafood byproducts. We review optimization strategies used to extract bioactive molecules from seafood byproducts. The parameters considered for bioactive extraction techniques and types of seafood byproducts are identified and their methods of optimization are reviewed. Limitations of the statistical optimization strategies are also analyzed and the best options are presented.

## 2. Statistical Optimization Strategies Applied for the Extraction of Bioactive Molecules from Seafood Byproducts

To study the effects of more than one treatment of an experiment, the experiment should be designed considering the following stages: (1) choosing and understanding the measuring instruments; (2) selecting the experimental subject; (3) selecting procedures and operations of the expected measurement. These stages are incorporated into the basic steps in designing the experiments: first, defining the problem expected to be solved; second, listing and understanding the factors that affect the extraction process; third, screening the factors that affect interactively by experimentation; last, optimizing the extraction process using the chosen factors. The optimized extraction condition should show efficient and quality yield at a lower extraction processing cost and time [18,19]. Therefore, statistical experimental design (DOE) methodologies are very important to obtain the required efficient amount of information at the lowest number, cost, and time of experimental analysis. This can be achieved by planning the testing method, applying appropriate data analysis, analyzing of interactive variability of factors, and reporting data in a scientific approach [18].

### 2.1. Classical Versus Multivariate Optimization Techniques Applied for the Extraction of Bioactive Molecules from Seafood Byproducts

Classical/univariate statistical optimization approaches are applied for comparing the means between two groups of analysis or to discriminate the effect of extraction variables using statistical analysis such as variance (ANOVA), t-tests, and Fisher’s multiple comparisons test. Moreover, statistical methods have been applied for the determination of the normality of the data and to detect outlier values during parameter testing and optimization [20,21]. Kumar et al. [1] studied chitinase production from shrimp waste using submerged fermentation. In this study, the fermentation variables screened using the Plackett–Burman method were incubation time, different media, pH, temperature, carbon source, nitrogen source, and metal ions. Equation (1) is applied to determine the effect of every factor on enzyme activities. Univariate statistical optimization techniques are dominantly applied for screening determinant extraction variables.

(1) $$E_{i}=\frac{\sum P_{i+}-\sum P_{i-}}{N}\;$$

where *E_i_* represents the effect of parameter *i* studied; *P_i+_* and *P_i−_* correspond to the responses of trails at which the parameter was at its high and low level, correspondingly; and *N* refers to the total number of trails. However, this time optimization approach has drawbacks (consume time and cost) when utilizing a large number of variables. It also has limitations on understanding the interaction effects of independent variables on the responses. However, it has been applied for optimizing bioprocesses to extract different active secondary metabolites [22].

The coupling of RSMs with statistical experimental design such as Doehlert design, full factorial design, BBD, and CCD was mostly used for optimizing extraction parameters, rather than other methods, as will be shown later. These experimental designs are applied for screening independent factors, selecting appropriate regression models, coding and defining the level of variables, verifying the fitted model, visualizing the predicted model equation, determining the optimal extraction condition, and validating the model equation (by measuring the response at the predicted optimal conditions).

### 2.2. Screening Extraction Parameters Used for the Extraction of Bioactive Compounds from Seafood Byproducts

During the definition of the problem expected to be solved and understanding and listing of the factors that affect the extraction process, intensive potential factors that affect the desired response may be present. These factors should be reduced by eliminating less important ones to save processing time and costs. Moreover, the level of complexity of the experimental designs can cause difficulties and experimental errors in understanding the interactive effects of the independent variables on the expected response. Selecting influencing factors by minimizing the number of experiments helps to collect the maximized information [18,23]. During parameter screening, the experiment should be based on the following: first, the need for the screening design should be identified; second, a specific number of the runs considering the range between the information gained and the extraction cost should be identified; last, feasibility and listing of the variables should be performed [18]. Some statistical software packages could give the screening outputs, depending on the researcher’s existing knowledge of the system and the extraction cost of factors. Extraction factor screening can be applied using Plackett–Burman design or fractional factorial if the factors are more than 5 and full or fractional factorial designs for a lower number of factors (2–4) [19]. To develop the fractional factorial design, the quantity of experimental points is calculated as *j^k^*^−1^, where *j* represents the number of factors to be tested and *k* is the number of levels. Multiple linear regression analysis should be applied to model the interaction between responses and the tested variables [24]. The readers can obtain details of the Plackett–Burman screening design from Vanaja and Shobha Rani [18]. The Plackett and Burman (PB) design is effective for screening *n* factors with *n* + 1 experiments (i.e., to screen seven variables, eight experiments should be conducted). It has been applied in designing chemometric tools combined with BBD. This screening design is only effectively applied to expected linear (main) effects, which do not consider factors in the interaction. Factors that significantly affect the response values are depicted using the Pareto chart of standardization effects as quality tools [25]. Nidheesh and Suresh [26] studied the optimization of chitin extraction from shrimp processing raw byproducts. They employed fractional factorial design as a factor screening technique and CCD coupled with RSMs to optimize the screened interaction effects of two variables. In particular, variables such as the concentration of HCl (%, *v*/*v*), reaction time (h), solid–liquid ratio of the HCl solution (*w*/*v*), and number of treatments were assessed for studying shrimp byproduct demineralization effects. They screened these variables into two categories based on their significant effect on the responses. Then, they optimized the effect of these two significant factors (concentration of HCl (%, *v*/*v*) and solid–liquid ratio of the HCl solution, *w*/*v*) on the demineralization process. The one-variable-at-a-time approach is applied to screen factors that affect the extraction of bioactive molecules. In particular, factors were screened that affect the deproteinization and demineralization during the extraction of chitin and chitosan from shrimp (*Parapenaeus longirostris*) shell waste. To achieve the highest deproteinization and demineralization degree, one-variable-at-a-time screening on the effects of carbon source (sucrose, glucose, or fructose) type, carbon source concentration, shrimp shell waste concentration, and incubation time were conducted before the optimization of the selected variables. Factors such as sucrose concentration, shrimp shell waste concentration, inoculum size, and fermentation time were selected for the optimized deproteinization and demineralization of the best chitin and chitosan extraction yields [27]. Ismail [28] applied a two-phase optimization model. They employed Plackett–Burman design as the first phase to screen multiple fermentation parameters that have the highest influence on the extraction of thermostable chitosanase and chitooligosaccharides from marine shrimp processing raw byproducts. Then, they employed BBD to optimize the screened variables (fermentation period, %MgSO_4_, and %KCl) for better chitosanase extraction yield. Seven independent factors named fermentation time temperature, period of microwave pretreatment of SPB, K_2_HPO_4_, MgSO_4_, KCl, and FeSO_4_·7H_2_O in eight experimental runs were screened in this study. The linear effect of the variables on chitosanase production was calculated using Equation (2).

(2) $$Y=B_{0}+\sum B_{i}X_{i}\;$$

where *Y* refers to the response value or chitosanase production, *B*_0_ represents the model intercept *B_i_* for the linear coefficient, and *X_i_* represents the level of the independent factor.

Taguchi design is also effective for screening significant extraction factors that affect the quality and yield of bioactive molecules. Many of these factors are screened and optimized for the best extraction of phytochemicals, total phenolic content, and antioxidant activity [29]. Moreover, Taguchi analysis has been applied to screen suitable and efficient extraction methodologies such as maceration, decoction, and microwave-assisted extraction [30]. Jabeur et al. [31] employed Taguchi experimental design to screen the most influencing factors named temperature, inoculum size of strain, and culture volume from nine factors to develop an optimized protease production. Similarly, a two-factor Taguchi orthogonal array was employed to optimize the oil extraction process from catfish heads. In this study, extraction temperature and time were screened as influential variables for better oil recovery and yield [32].

### 2.3. Screening Used for Selecting Potential Extraction Solvents and Hydrolyzing Enzymes

The different extraction capacities of the bioactive molecules are presented for individual polar and non-polar solvents. However, the mixtures of the polar and non-polar show better extraction. Hence, screening of these appropriate solvents for selecting potential extraction solvents is very important. Moreover, solvents like microemulsion (containing tributyloctylphosphonium bromide, tributyloctylphosphonium trifluoroacetate, or tetrabutylphosphonium trifluoroacetate) have stronger electrostatic and hydrogen bonding interactions than the less-polar solvents (ethanol, acetone, and dimethyl sulfoxide), which can enhance the extraction of bioactive compounds such as astaxanthin [33]. In a study conducted to extract astaxanthin from shrimp (green tiger, *Penaeus semisulcatus*) shells using ultrasonic-assisted extraction, the individual effects of solvents (petroleum ether, n-hexane, ethanol, and acetone) and ternary mixtures of petroleum ether, acetone, and water were screened. The solvents with higher polarity were reported to be the most effective for astaxanthin extraction. Moreover, the effect of different ternary mixtures of petroleum ether/acetone/water solvents has been shown for larger extractions of astaxanthin. This is due to the fact that, during the extraction of bioactive molecules, the solvents can diffuse into the material substrate and dissolve molecules that have relative polarity; however, the non-polar solvents withhold from diffusing into the hydrophilic layer [34]. During the production of protein hydrolysates from undersized hakes (fish bycatch), enzymatic activities of broadspectrum endoprotease, serine-type endoprotease, trypsin-specific protease, chymotrypsin-like protease, blend of endo- and exopeptidases, and glutamic-acid-specific protease were screened to select the best hydrolyzing enzyme [35].

### 2.4. Multivariate Regression Model Selection and Optimization of Screened Extraction Parameters of Bioactive Compounds

Once the determining extraction parameters/variables are screened, selecting an appropriate statistical regression methodology to study their interaction with the dependent variables is crucial [19,36]. From the study of the relationship between independent and dependent variables, it is possible to show if the model can be linear, quadratic, or cubic with coefficients that indicate values and signals that help to interpret the influence of the factors. Multivariate statistical regression methodologies such as RSMs, the non-linear least-squares (quasi-Newton) method [37,38,39], the particle swarm optimization algorithm [40,41], and artificial neural networks (ANNs) [40] were employed. Some of the statistical regression methodologies were applied in combination with two or three methods such as the particle swarm optimization algorithm with an artificial neural network [40] and an RSM with the Genetic algorithm and particle swarm [41]. In particular, for the extraction of chitinase from shrimp shell waste, the chitinase activity was optimized using a particle swarm optimization algorithm and artificial neural network by controlling the variables (colloidal chitin, glucose, Tween 80 (common surfactant micelles), and yeast extract) of the fermentation medium [40]. Sharayei et al. [34] studied optimizing extraction variables using the ultrasonic method employing RSMs. First, they optimized the effect of solvent type and extraction time. Then, they adjusted the extraction temperature, extraction time, and ultrasound amplitude to optimize astaxanthin extraction efficiency from shrimp (green tiger, *Penaeus semisulcatus*) shells. In their study, they suggested that the green extraction method (applying ultrasonication) is safe and efficient compared to the non-polar solvent (petroleum ether and n-hexane) extraction of astaxanthin pigment with higher antioxidant activity.

Non-linear least-squares (quasi-Newton), the particle swarm optimization algorithm, and artificial neural network (ANN) optimization methods have rarely been employed to optimize the extraction parameters of bioactive compounds from seafood byproducts. Vázquez et al. [37] optimized proteolytic digestion independent variables (pH, temperature, and protease concentration) for protein hydrolysate production from monkfish (*Lophius piscatorius*) heads and viscera using the non-linear least-squares (quasi-Newton) method. From the calculated individual percentage contributions (PC) of independent variables using Equation (7), the quadratic terms (pH and temperature) of the developed models have shown a significant effect on the enzyme proteolysis of monkfish. Moreover, an enzymatic hydrolysis optimization study was conducted by controlling temperature and pH as critical factors to produce protein hydrolysates from *Scyliorhinus canicula* discards (muscle) [38]. From the developed equation quadratic term for the alcalde enzyme, hydrolysis (95.8%) and the linear effect (temperature, 97.2%) of esterase enzyme hydrolysis have shown the highest percentage contributions compared to the other terms.

The particle swarm optimization method is applicable for optimizing complex optimization problems, such as fermentation process parameters that were developed by Kennedy and Eberhart [42]. This method is applicable for searching for the best values by linking and exchanging knowledge among swarm individuals. In particular, Suryawanshi and Eswari [40] studied the production of chitin from seafood byproducts like shells, tails, heads, and bones via enzyme hydrolysis optimized using the particle swarm optimization algorithm and artificial neural network optimizations considering colloidal chitin, glucose, Tween 80 (common surfactant micelles), and yeast extract as basic fermentation medium factors.

The Genetic algorithm as part of randomized search optimizations (natural evolution studies) is applicable for presenting initial conditions in previously developed process mathematical model. It has been applied for optimizing protein extraction in an aqueous two-phase system [41,43]. Saravana Pandian et al. [41] conducted an aqueous two-phase system protein extraction yield from shrimp (*Litopenaeus vannamei*) waste. They optimized the process condition considering polyethylene glycol concentration, trisodium citrate concentration, pH, and temperature as determining factors. In their study, they employed the RSM-coupled Genetic algorithm (GA) and particle swarm. The RSM-optimized parameters were used as initial conditions for the Genetic algorithm partitioning study of the recovered proteins. Moreover, the initial conditioning of the RSM regression equation was utilized for studying parameter influences over the process using particle swarm optimization. From the developed optimization models of the top-phase protein yield response, the calculated maximum percentage contributions of the terms are from the linear (59.5%) and the quadratic (40.1%).

#### 2.4.1. Response Surface Optimization (RSM) as a Tool to Optimize the Extraction Parameters of Bioactive Compounds

RSMs are a collection of mathematical and statistical techniques where experimental data are fitted using a polynomial equation. It is applicable to show the effect of independent factors on the dependent (response) variables using a generated empirical model. Moreover, it is a more suitable methodology to select if the extraction processing data favors a linear or square polynomial function. An RSM is applied by coupling it with different experimental designs such as Doehlert design, full factorial design, BBD, and CCD [19,22,36]. Most of the multivariate statistical optimizations employed for optimizing the extraction parameters of bioactive compounds from seafood byproducts were RSM-coupled Box–Behnken and CCDs.

##### Choice of the RSM Experimental Design

The choice to apply RSM-coupled experimental designs (Doehlert design, full factorial design, BBD, or CCD) is mediated by the applicability (efficiency of parameters) for a larger number of experiments, number of experiments/runs and blocks, required number of variables/factor level used, center point used, selection of experimental points, and axial points used. The three-level factorial design is not efficient if the number of factors is greater than 2 [19,44].

Suitable models starting from a linear function (simplest model), as shown in Equation (3), should be tested against the obtained responses. In this linear model, the responses should not show any curvature. Any curvature observed should be evaluated using a second-order model that has central points. Interaction effects between experimental variables are evaluated by applying polynomial models (Equation (4)) that have additional terms. Critical points (maximum, minimum, or saddle) of the variables are evaluated using a polynomial function (Equation (5)) that contains quadratic terms. Moreover, this polynomial function should be performed using at least three factor levels. BBD, three-level factorial design, CCD, and Doehlert design are commonly applicable second-order symmetrical designs [19,36].

(3) $$y=\beta_{0}\sum_{i=1}^{k}\beta_{i}x_{i}+\varepsilon$$

where *k* represents the number of variables, *β*_0_ is the constant term, *β_i_* represents the coefficients of the linear parameters, *x_i_* represents the variables, and *ε* refers to the residual associated with the experiments.

(4) $$y=\beta_{0}+\sum_{i=1}^{k}\beta_{i}x_{i}+\sum_{1\leq i\leq j}^{k}\beta_{ij}x_{i}x_{j\;}+\varepsilon$$

(5) $$y=\beta_{0}+\sum_{i=1}^{k}\beta_{i}x_{i}+\sum_{i=1}^{k}\beta_{ii}x_{i}^{2}+\sum_{1\leq i\leq j}^{k}\beta_{ij}x_{i}x_{j\;}+\varepsilon$$

where *β_ij_* and *β_ii_* refer to the regression coefficients of interactive parameters and quadratic parameters, respectively.

##### Coding the Factor Levels

The most important reason to codify the factor levels is to avoid statistical weighting of the factors due to the differences between the numerical range and values of the variables. Factors with different units and levels must be coded by converting their real value into ranges by keeping their dimensions (−1 to +1) when the design is developed based on the coded value. The real *Z_i_* value can be changed to coded values *x_i_* using Equation (6). Defining the level of factors is very critical for the achievement of process optimization of the screened variables before conducting the regression analysis, which also helps for codification [36].

(6) $$x_{i}=\frac{Z_{i}-Z_{i}^{0}}{∆Z_{i}},\;i=1,2,\ldots \ldots ,k$$

where *x_i_* refers to the dimensionless coded value of the independent factor, *Z_i_* corresponds to the actual value of the independent factor *i*, *Z*^0^*_i_* refers to the real value of the independent factor at the center point, and ∆*Z_i_* refers to the step change of the real value in the center point. Some studies on the optimization of the extraction of bioactive compounds from seafood byproducts in the present review applied coding of the factor levels. However, others studies conducted by Blanco et al. [45], Srinivasan et al. [46], and Tsiaka et al. [47] used both the coding and actual values for better clarity.

##### Central Composite Design (CCD)

Many studies applied CCD coupled with RSM to optimize the extraction parameters of bioactive compounds from seafood byproducts. The CCD is applicable for sequential experimentations with reasonable evidence since it contains three-point types: (1) full factorial or fractional factorial design; (2) a central point; (3) axial points. The axial points are additional designs to show if the experimental points are at some distance from the center point. Complete routable CCDs are characterized as follows: (1) Experimental numbers should be calculated as *N* = 2*^k^* + 2*k* + *c_p_*, where *k* is the number of extraction process factors, 2*^k^* is the number of designed factorial points, 2*k* the number of axial points at a distance of ±α, and *c_p_* is the replicate number of the central point. (2) Considering the number of variables, the α/axial points should be calculated as α = 2 ^(k)1/4^. (3) Factors should be investigated at five levels (−α, −1, 0, +1, and +α) [36,48].

Nidheesh and Suresh [26] studied the optimization of isolation conditions of high-quality chitin from shrimp byproducts. In their optimization, they applied a two-level, center point fractional factorial design (FFD) for identifying influential shrimp byproduct demineralization variables (concentration of the HCl solution, reaction time, solid–liquid ratio of the HCl solution, and number of treatments). Similarly, for the deproteinization of demineralized shrimp byproducts they screened five variables (reaction time, solid–liquid ratio of the NaOH solution, and number of treatments as before and then adding two new variables—reaction temperature and concentration of the NaOH solution). Then, they optimized the screened variables for demineralization (concentration of the HCl solution and solid–liquid ratio of HCl solution) and deproteinization of demineralized (concentration of the NaOH solution, reaction temperature, and solid–liquid ratio of NaOH solution) of shrimp byproducts using CCD. Some RSM coupled with CCD-fitted/developed models, which were conducted on the extraction of bioactive compounds from seafood byproducts, were selected to investigate their effect on the response or extraction yields. The individual percentage contributions (PC) of extraction variables from such methodologies can be calculated using the Equation (7) [48,49,50]. The total percentage contributions (TPC) of linear, quadratic, and interactive terms of the selected independent variables were calculated using Equations (8)–(10) for better understanding of the effect of extraction variables on yield/response [51,52].

(7) $${PC}_{i}=\left(\frac{\beta_{i}^{2}}{\sum \beta_{i}^{2}}\right)\times 100\;(i\neq 0)$$

where *β_i_* represents the regression coefficients of each individual extraction process. This equation is preferable for screening extraction variables, which can be visualized using a Pareto chart [51,52].

The total percent contribution of the linear, quadratic, and interactive terms of extraction variables can be calculated using the following equations [49]:

(8) $${TPC}_{i}=\frac{\sum_{i=1}^{n}{SS}_{i}}{\sum_{i=1}^{n}\sum_{i=1}^{n}{SS}_{i}+{SS}_{ii}+{SS}_{ij}\;}\times 100,$$

(9) $${TPC}_{ii}=\frac{\sum_{i=1}^{n}{SS}_{ii}}{\sum_{i=1}^{n}\sum_{i=1}^{n}{SS}_{i}+{SS}_{ii}+{SS}_{ij}\;}\times 100,$$

(10) $${TPC}_{ij}=\frac{\sum_{i=1}^{n}{SS}_{ij}}{\sum_{i=1}^{n}\sum_{i=1}^{n}{SS}_{i}+{SS}_{ii}+{SS}_{ij}\;}\times 100$$

where TPC*_i_*, TPC*_ii_*, and TPC*_ij_* refer to the total percentage contributions (TPC) of linear, quadratic, and interactive terms; SS*_i_*, SS*_ii_*, and SS*_ij_* represent the computed sum of square (SS) of the linear, interactive, and quadratic terms, correspondingly.

The calculated total percentage contributions (TPC) of linear (88.8%), quadratic (16.2%), and interactive (3%) terms of the variables (concentration of the NaOH solution, reaction temperature, and solid–liquid ratio of the NaOH solution) for the deproteinization of demineralized shrimp byproducts show that their individual activities are more influential than their quadratic and interactive terms. In another optimization study on the microwave-assisted extraction of nutritional oil yield from fish heads and fins, the linear (88.7%, 51.2%) terms dominantly affected the extraction yield, rather than the quadratic (6.8%, 47.6%) and interactive (4.5%, 1.2%) terms of the total percentage contributions (TPC) of variables (time, microwave power, and solid–liquid ratio) [3]. Similarly, Blanco et al. [45] analyzed the impact of chemical treatment (NaOH concentration), temperature, time, and concentration of acetic acid (AcOH) on the extractability of skin collagen from Small-Spotted Catsharks. Two experimental designs, one for each of the main stages of the process, were achieved using RSMs. The combined effect of NaOH, time, and temperature on the amount of collagen recovered in the first stage of the collagen extraction procedure was investigated. Secondly, skins treated under optimal NaOH conditions were exposed to a second experimental design, to study the combined effect of AcOH concentration, time, and temperature on collagen recovery using yield, amino acid content, and SDS-PAGE characterization. In this study, the linear (86.5%) effect of the variables is more significant than their quadratic (13.5%) effects. The calculated total % contributions of the linear and quadratic terms of the developed model are more influential than their interactive terms. The values of independent variables maximizing collagen recovery were 4 °C, 2 h, and 0.1 M NaOH (as the pretreatment) and 25 °C, 34 h, and 1 M AcOH (for collagen extraction).

In the other study conducted by Pinela et al. [3], the potential of the microwave-assisted extraction (MAE) technique to obtain high-quality oil from fish byproducts was explored. The independent variables, time (1–30 min), microwave power (50–1000 W), and the solid–liquid ratio (70–120 g/L), were joined in a 20 run experimental design coupled with an RSM. To establish the theoretical models, values of yield oil were fitted to a quadratic equation. The yielded oil was expressively affected by the three independent variables through linear, quadratic, and/or interactive effects. Compared to a conventional Soxhlet extraction (SE), the optimal MAE conditions allowed between 60 and 100% of oil to be recovered in less than 19 min and with less solvent consumption. In addition, these oils were mainly constituted by oleic, docosahexaenoic (DHA), linoleic, and eicosapentaenoic (EPA) acids.

**Figure 1:**
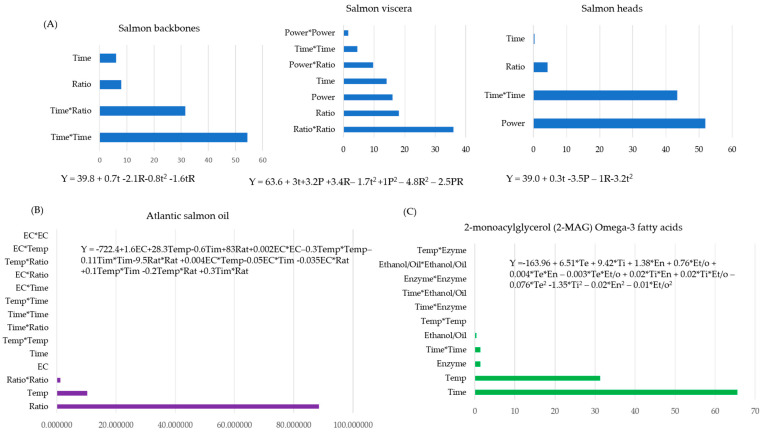
Individual percentage contributions (PC) of independent variables to the optimization of bioactive compound extraction from seafood byproduct: (**A**) *Salmon viscera*, backbones, and heads using CCD; (**B**) Atlantic salmon waste employing FFD; (**C**) Atlantic salmon (*Salmo salar*) bone using BBD [53,54,55].

Optimizations models of fish bioactive oil extraction yield considering time, microwave power, and solid–liquid ratio from salmon viscera, salmon backbones, and salmon heads using microwave-assisted extraction were developed by de la Fuente et al. [53]. In this study, the effect of quadratic terms (time and ratio) are more influential than their interactive terms for optimizing theoil extraction yield from salmon viscera, backbones, and heads (Figure 1A). However, the linear term of the effect of microwave power shows greater influence on the extraction of oil from salmon heads.

##### Box–Behnken Design (BBD)

Box–Behnken design is a three-level arrangement of factors that can be applicable for estimating the coefficients of the first- and second-order mathematical models. Mainly, it contains a particular subset that originates from factorial combinations of the *3^k^* factorial design. It is a more efficient and economical experimental design utilized for designing larger variables. In this design, the experimental points are equally distant from the center point, which requires the following: (1) Experimental numbers calculated using the equation *N* = 2*k*(*k* − 1) + *c_p_*, where *k* represents the number of factors and (*c_p_*) the number of center points. (2) Factor levels should have equally spaced intervals and be arranged only into three-levels (−1, 0, and 1). The three levels are low (−), high (+), and control or basal points in which the extreme points between factors or their high-level factors involved in the process can be evaluated [19,36].

The most influential independent variables selected to optimize the production of chitosanase and chitooligosaccharides from marine shrimp processing raw byproducts using a solid-state fermentation of enzyme production were the fermentation period, concentration of MgSO_4_, and concentration of KCl [28]. From this study, the total percentage contribution of the linear and quadratic terms of the developed model is the most influential. Moreover, to study the improvement in chitosan production from Indian white shrimp waste using chemical and microwave methods, the basic parameters optimized were the temperature, concentration of alkaline, time of chemical reaction, power of the microwave, irradiation time, and concentration of alkaline for the microwave method. From the regression models developed to predict the effect of the variables, the linear and quadratic terms of both models are the greatest total percentage contributors to the chitosan yield. But, at an elevated microwave power and longer heating times, the yield may decrease due to the inhibition of the deacetylation reaction of chitosan [56]. Chandra Roy et al. [57] investigated the extraction of astaxanthin from shrimp (*Penaeus monodon*) shells using ultrasound-assisted natural deep eutectic solvents. The extraction process was optimized considering the natural deep eutectic solvent molar ratio, ultrasound amplitude, and extraction time as basic independent variables. From their developed model the extraction yield of astaxanthin was affected dominantly by linear and quadratic terms. The ultrasonication power and sonication time factors strongly influence the extraction yield, which could be inhibited at an elevated level. Optimization of the enzymatic hydrolysis reaction for the production of protein hydrolysate from scallop (*Argopecten purpuratus*) visceral meal and defatted meal was conducted to study the effect of process variables such as temperature, time, and enzyme concentration (enzyme/substrate level). The total contribution of factors in linear terms was more influential than the quadratic and interactive terms [58]. In a supercritical carbon dioxide extraction of oil enriched with eicosapentaenoic acid and docosahexaenoic acid from Atlantic salmon frame bones, the effects of important variables such as the urea/fatty acids ratio, crystallization temperature, and crystallization time were optimized [59]. From the prediction regression model, the linear term has shown a greater total percentage contribution than its quadratic and interactive terms. From this study, it is reported that the urea to fatty acid ratio is the most influential factor due to its contribution to the urea complexation process. In another study conducted on the production of carotenoids from Red shrimp (*A. antennatus)* heads using ultrasound-assisted and microwave-assisted extractions, the basic processing variables optimized were extraction time, ultrasound and microwave power, and the solvent/material ratio. The carotenoid extraction yield obtained using the two modern extraction methods was affected by the quadratic linear and interaction terms. In particular, the total percentage contribution of the quadratic term dominantly contributed to the ultrasound-assisted extraction, whereas the microwave-assisted extraction is affected by the interactive terms rather than the linear terms [47]. These authors reported an improvement in the extraction efficiency of carotenoid compounds using UAE and identified the optimal extraction conditions as follows: ultrasound exposure time: 5 min; ultrasound power: 600 w; mixing ratio: 1:20 mL/g with acetone solvent. In addition, they stated that the UAE method is faster, easier, and more reproducible than conventional extraction methods. The summarized total percentage contribution of variables that affect the production of bioactive compounds from seafood byproducts that are optimized using RSM-coupled BBD are presented in Table 1. The extraction yield (2-monoacylglycerol) of 2-monoacylglycerol (2-MAG) omega-3 fatty acids from Atlantic salmon (*Salmo salar*) bones using the supercritical carbon dioxide method was optimized using RSM-coupled BBD, considering extraction variables such as reaction temperature, time, enzyme load, and the ethanol: oil molar ratio [55]. Individual percentage contributions of linear terms of the model equation developed show more influence than the interactive and quadratic terms, which is depicted in Figure 1C.

#### 2.4.2. Full Factorial Design

Full three-level factorial design is rarely applied in RSM optimization of bioactive molecules from seafoods byproducts compared to Box–Behnken, central composite, and Doehlert designs at factor numbers greater than two. This is due to the experiment numbers (*N*) required (which can be calculated as *N* = 3*k*, where *k* represents several factors) being very high, so modelling of the quadratic functions can be inefficient. Fractional factorial design is preferable when the number of variables is greater than two, which is mostly applied for screening larger variables [19,36].

Some studies for the extraction of bioactive compounds from seafood byproducts have applied the full three-level factorial design coupled with an RSM. In a study conducted on the natural deep eutectic solvent (choline chloride and L(+)-tartaric acid)-based ultrasound and microwave-assisted extraction of carotenoids from shrimp waste, RSM-coupled two- and three-level fractional factorial experimental designs were applied to study the effects of extraction variables such as extraction time, solvent-to-propolis, and the choline chloride: tartaric acid-to-H_2_O ratio on the carotenoid yield [60]. Moreover, Ramakrishnan et al. [54] studied the enzymatic transesterification optimization of biodiesel yield from Atlantic salmon (*Salmo salar*), considering crucial factors such as enzyme concentration, temperature, the oil/alcohol molar ratio, and time. The individual percentage contributions of the model linear terms (temperature and oil/alcohol molar ratio) are more significant than the quadratic and interactive terms (Figure 1B). They suggested that incorporating these terms into the developed model can make it unstable and difficult to interpret.

##### Doehlert Design

Doehlert design is considered to be practical and economical compared to other second-order experimental designs, which also require small experimental points to make them applicable and efficient. It is mainly characterized as follows: (1) The experiment number should be calculated using *N* = *k*^2^ + *k* + *c_p_*, where *k* refers to the number of factors, *c_p_* is the number of center-point replication. (2) Important considerations such as cost and/or instrumental constraints of variables can be studied at a major or minor number of levels. (3) Intervals can be uniformly distributed among levels. (4) Previous adjacent points can be used to displace the experimental matrix from another experimental region [36]. From our current study on statistical optimization strategies, none of the approaches to extracting bioactive compounds from seafood byproducts have applied an RSM-coupled Doehlert design. However, other studies on bioactive component extraction from other sources have been applied. For instance, da Silva Bambirra Alves et al. [61] studied the production of protein hydrolysates from chicken blood meal using enzymatic hydrolysis by optimizing critical factors such as temperature, pH, and the enzyme-to-substrate ratio while employing the Doehlert design matrix.

##### Presentation of the Model and Determination of Optimal Conditions

**Figure 2:**
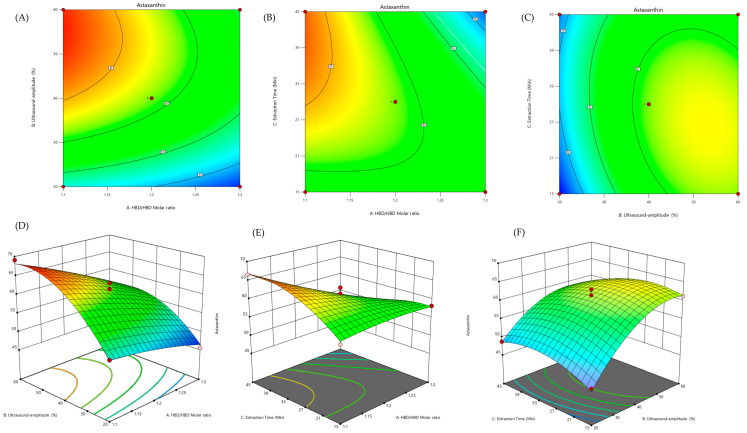
(**A**–**C**) are contour plots of two-dimensional plots and (**D**–**F**) are 3D surfaces representing the interaction effect of the natural deep eutectic solvents molar ratio (hydrogen bond donor HBD/acceptor HBA), ultrasound amplitude, and extraction time on the ultrasound-assisted astaxanthin extraction yield from shrimp shells [57].

Surface and response contour plots are theoretical two- and three-dimensional outputs mostly utilized to visualize the predicted model equations and depict the relationship among the dependent and independent variables. These are also applied to show any changes in the independent factors that lead to changes in the response values. Where contour plot works on a two-dimensional surface plot, this improves our understanding of the plots of the response surface. When the contour plot shows ellipses or circles then the experimental region is in the maximum, minimum, or ranged point; however, if the contour plot depicts parabolic or hyperbolic shapes the target point is saddle point or not maximum nor minimum. Moreover, surface responses (3D surfaces) generated from a quadratic model in the optimization of two variables are important to show a more realistic visualization of optimum points [19,36,62]. The ellipses-shaped contour plots developed from an optimization process of ultrasound-assisted astaxanthin extraction yield from shrimp shells are shown in Figure 2A–C. Similarly, Figure 2D–F depicts two independent variables optimized for the extraction of astaxanthin yield from shrimp shells. According to Figure 2, the predicted model was expressed using extraction parameters: hydrogen bond donor (HBD)/acceptor (HBA) molar ratio (CC/LA 1:1.02); ultrasound amplitude (54.43%); and extraction time (39.23 min). The predicted regression coefficient and expected yield (69.09 µg/g of shrimp waste) are close to the actual yield (68.98 µg/g of shrimp waste). These results revealed that ultrasonication power and sonication time proved to be significant factors for the extraction of astaxanthin yield [57].

A three-dimensional plot of two-dimensional representation is plotted using statistical software packages such as Design Expert (version 7.0.0-10.0), Sigma Plot (Sigmaplot-11), SPSS (Version 11.0.1.2001), and STATISTICA (Version 10) for graphical representation/visualization of fitted model equations [56,63]. So, for more than three independent variables, the plot visualization is only applicable when one or more variables will be set at a constant value. There should be a consideration that the response surface and contour plots only show the estimated response and the general nature of the optimization system from the fitted model but not the true structure. Although limited multivariate optimization strategies listed have used response surface polynomials to locate the maximum or minimum effects of independent variables, many of them have demonstrated response surface plots. The response surface graphs are not sufficient to locate the maximum or minimum value. On the contrary, one must work directly with the response surface polynomials and find the maximizing or minimizing factors. Hence, other methods involve computing the first derivative of the fitted model function equal to zero then finding the stationary point by solving the linear equations. If the fitted model equation is like Equation (11) [19,62],

(11) $$Y=\beta_{0}+\beta_{1}X_{1}+\beta_{2}X_{2}+\beta_{11}X_{1}^{2}+\beta_{22}X_{2}^{2}+\beta_{12}{X_{1}X}_{2}.$$

By computing the first derivative ($\partial Y/{\partial X}_{1}$) and ($\partial Y/{\partial X}_{2})$ of this equation and setting zero, one can find the stationary point from Equations (12) and (13). These equations can be solved using the Excel Solver tool.

(12) $$\frac{\partial Y}{{\partial X}_{1}}=\beta_{1}+{2\beta }_{11}X_{1}+\beta_{12}X_{2}=0\;$$

(13) $$\frac{\partial Y}{{\partial X}_{1}}=\beta_{2}+{2\beta }_{22}X_{2}+\beta_{12}X_{1}=0\;$$

where *X*_1_ and *X*_2_ refer for the coded values of the independent variables that give the highest or lowest response. Generally, the stationary point (minimum or maximum point) should be identified in the ranges of the tested independent parameters from the fitted second-order equation [62].

##### Robustness, Validation, and Verification of Predicted Models/Optimized Extraction Conditions

**Figure 3:**
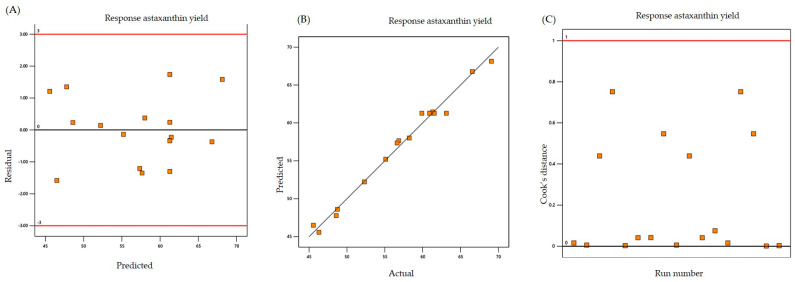
Diagnostic plots for the model adequacy developed for the extraction of astaxanthin yield from shrimp shells: (**A**) residual versus predicted values; (**B**) predicted versus actual values; (**C**) Cook’s distance versus run number.

Residual plots are valuable criteria to evaluate if the observed error (residuals) and stochastic error are consistent, in which the residuals should be centered on zero within the fitted values and should not be systematically high or low. Undesirable residual plotting (residual analysis) shows a non-random pattern in which the predictor variables in the fitted model indicate the possibilities of missing variables and/or the presence of curvature due to higher-order terms of variables [19]. Figure 3 shows a residual plot of an ultrasound-assisted astaxanthin extraction yield from shrimp shells in which the residuals are slightly scattered from the center point and the residuals are constantly spread throughout the range. Moreover, model adequacy can be evaluated by plotting predicted versus actual values (Figure 3B) and Cook’s distance values versus run number (Figure 3C) [57]. The plot for predicted versus actual values shows the points of all predicted and experimental response values present very close to the 45° line as there is a correlation between the influence of the process variables on the response of the developed model. Similarly, Cook’s distance values fall in the determined range indicating the experimental data have no strong evidence of influential error observations. Studies conducted on the optimization of oil from aqueous, two-phase protein extraction from *Litopenaeus vannamei* waste [41], oil enriched with eicosapentaenoic acid and docosahexaenoic acid extraction from Atlantic salmon byproduct oil [59], the production of omega-3 fatty acids (rich 2-Monoacylglycerol) from Atlantic salmon oil byproducts [55], chitosan production from Persian Gulf shrimp waste [56], and the extraction of high-energy carotenoid from *Aristeus antennatus* shrimp [47] used residual plots to check the models for any undesirable residuals.

Analysis of variance (ANOVA) is more reliable way to evaluate the statistical significance of a developed model by applying descriptive statistical analysis such as the standard deviation, prediction error sum of squares (PRESS) residuals, the lack-of-fit test, the coefficient of variation, the coefficient of determination (R^2^), the adjusted determination coefficient (adj-R^2^), adequacy precision, the F-value, and the *p*-value. Moreover, analysis variance using the F–Fisher test employing the different mean square ratios (F_1_ = Model/Total error, F_2_ = (Model + Lack of fitting)/Model, F_3_ = Total error/Experimental error, and F_4_ = Lack of fitting/Experimental error) could be applied to confirm the robustness and significance of the empirical equation. These ratios are essential to avoid type-I and type-II errors [64]. A mathematical model has been accurately fitted to the experimental data when the mean square of regression *lack of fit test* reflects only the random errors inherent to the system. Moreover, the mean square of the regression predicted error is the estimate of these random errors, and it is assumed that these two values are not statistically different [36]. The correctness of the model with experimental data can be evaluated via the adequacy of precision, determination coefficient R^2^, and adj-R^2^. An adequate model is explained showing that the difference between the adj-R^2^ and predicted R^2^ (Adj-R^2^–Pre-R^2^) should be less than 0.2, with maximum PRESS, and with a predicted R^2^ value greater than 0.7. Adequacy precision measures the signal-to-noise ratio in which a ratio greater than 4 is desirable [36]. However, verification of the adequacy of the fitted model using the above statistical analysis only is not sufficient. There are two reasons that the coefficient of determination (R^2^) alone cannot show the accuracy of the model. First, it will increase when the number of contributing variables to the model increases, neglecting the statistical significance of the added variable. Second, measurement of the decreasing changeability of the achieved responses applying the affecting variables in the model is depicted by the R^2^ index. Hence, the accuracy of the model should also be checked using absolute average deviation (AAD) (Equation (14)), showing statistical dispersion or variability or the central point’s absolute deviations [65]. From the analysis of R^2^ and AAD, it is expected that the R^2^ must be near to 1 and the range of estimated and observed AAD must be as low as possible [66].

(14) $$AAD\;(\%)=\left\{\left[\sum_{i=1}^{p}\left(\frac{\left|{yi}_{exp}-{yi}_{cal}\right|}{{yi}_{exp}}\right)\right]/p\right\}\times 100$$

where *p* indicates the number of experiments as well as *yi_exp_* and *yi_cal_* for experimental and calculated outputs of the experimental results, respectively. The reference results of the analysis of variance (ANOVA) for the regression model developed and the calculated AAD for the extraction of astaxanthin yield from shrimp shells are summarized in Table 2. The calculated AAD (%) presented in Table 2 gives additional adequacy information for the developed response. Most of the studies reviewed here considered ANOVA to discriminate the model developed. Although the AAD is a very important criterion to evaluate the adequacy/suitability of fitting the response surface of a model, no statistical optimization strategies in the current review were applied to verify the adequacy of the developed models for the extraction of bioactive molecules from seafood byproducts.

Fitting experimental data, analyzing the data, checking the validity of the fitted model, and determining the optimum extraction conditions are not enough to publish the adequacy of the developed model. Conducting confirmation experimental works at the optimized factor values and comparing the mean data with predicted values is very important for checking the reliability of the process. To calculate the significance of coefficients from the polynomial equations obtained after fitting experimental and calculated data from the corresponding factorial designs, Student’s *t* test must be applied. Unfortunately, limited studies under this review considered conducting two or more confirmation experimental works under the selected optimum conditions.

Table 3 shows the application of univariate statistical strategies for screening and optimizing bioactive molecule extractions from seafood byproducts, whereas Table 4 shows multivariate techniques of statistical optimization strategies applied for the extraction of bioactive molecules from seafood byproducts.

## 3. Extraction Process Parameters Considered for Bioactive Molecules from Seafood Byproducts

### 3.1. Chitin and Chitosan

Extracting chitin and chitosan from discarded seafood skeletons requires particular attention to multiple factors to ensure maximum output and effectiveness. Determining the suitable extraction method using chemical, physical, or biological is crucial, though each method has its own set of advantages and drawbacks [81,82].

Variables like the type of seafood waste (like shrimp or crab shells), their size, and composition significantly impact the extraction process. Parameters like temperature, pH, and the duration of the reaction are pivotal in chemical and enzymatic extraction techniques, affecting both the rate of chitin breakdown and impurity elimination. Moreover, careful selection of demineralization and deproteinization agents, whether solvents, acids, or alkalis, is imperative to achieve chitin of high purity. The selection of a demineralization agent significantly impacts the effectiveness of mineral removal, whereas the deproteinization agent plays a crucial role in eliminating proteins without compromising chitin integrity. In addition, variables such as the ratio of waste material to extraction solvent, agitation speed during processing, and the incorporation of co-solvents can also influence both the efficiency and quality of chitin and chitosan extracted from seafood byproducts. Some of the extraction parameters considered during the extraction of chitin and chitosan are summarized in Table 4.

The extracting process of chitin and chitosan employed one variable at a time and/or multivariate optimization strategies. For instance, the one-variable-at-a-time optimization method was employed for the production of chitinase from shrimp waste using submerged fermentation. In this method, the effect of parameters such as incubation time, different media, pH, temperature, carbon source, nitrogen source, and metal ions were screened using the Plackett–Burman method [1]. Moreover, the extraction of chitin from shrimp shell waste and speckled shrimp *Metapenaeus monoceros* shells were optimized using RSM-coupled CCD and BBD, respectively [31,40]. In these methods, extraction parameters for the fermentation (colloidal chitin, glucose, Tween 80 (common surfactant micelles), and yeast extract) and hydrolysis (temperature, inoculum size of strain, and culture volume) were optimized.

Regarding utilizing fermentation for deproteinization, microbes can naturally occur within the chitosan source (auto fermentation) or be introduced into the source for deproteinization and/or demineralization. In these fermentation stages, deproteinization is achieved through proteolytic enzymes, while demineralization is facilitated by the organic acids generated by the microorganisms. For instance, for the production of chitin and chitosan from shrimp (*Parapenaeus longirostris*) shell waste the fermentation process was optimized by employing BBD coupled with an RSM. At the optimized fermentation process parameters (sucrose concentration = 5%; shrimp shells waste concentration = 12.5%; inoculum size = 10%, containing 35 × 10^8^ CFU/mL; incubation period = 7 days) the degree of deproteinization and demineralization was maximized to 75.27% and 63.50%, respectively [27].

Optimizing enzymatic hydrolysis parameters of chitin and chitosan production are important for efficient production. Chitin production from black tiger shrimp (*Penaeus monodon*) shells was optimized using BBD at an optimum pH (8.82), temperature (50.05 °C), agitation speed (100.98 rpm), enzyme–substrate ratio of 1:8 (*w*/*v*) and incubation period (72 h) [63].

Thermochemical treatments, chemical and microwave, are other extraction methods of chitin and chitosan production employing RSM optimization [26]. In particular, thermochemical treatments for the production of chitin from shrimp (*Penaeus* sp.) cephalothoraxes and carapaces were optimized as a dry and wet base using a CCD-coupled RSM. In this study, treatment variables such as the concentration of HCl (%, *v*/*v*) at 4.5 (for wet) and 4.9 (for dry), reaction time at 3 h, and solid-liquid ratio of HCl (*w*/*v*) at 1:5.5 (for wet) and 1:7.9 (for dry) were optimum for 98% demineralization of shrimp byproduct. Parameters such as the concentration of NaOH at 3.6% (*w*/*v*), reaction time at 2.5 h, temperature at 69 °C, and solid–liquid ratio of NaOH at 7.4 (*w*/*v*) were also optimum values for the 98% deproteinization of the demineralized byproducts [26]. The production of chitosan from Indian white shrimp waste was optimized employing BBD and was investigated by Nouri et al. [56]. They reported the optimum microwave-assisted extraction parameters as 50% NaOH solution, 720 W microwave power and 20 S reaction time. At these optimum points, the highest percent of chitosan preparation (19.47%) and degree of deacetylation (89.34%) were reported.

### 3.2. Proteins and Peptides

Enzymatic hydrolysis via endogenous enzymes (autolysis) present in the fish’s digestive system typically requires extended periods to generate substantial amounts of cleaved peptides [83,84]. Siddik et al. [83] highlighted the challenges in standardizing and controlling the autolysis process, as enzyme production depends on various factors like age, season, species, diet, and environment. Conversely, the utilization of commercial enzymes in enzymatic protein hydrolysis offers numerous advantages over autolysis or chemical hydrolysis. This might lead to improved functionalities and bioactivities, whereas autolysis might cause the accumulation of undesirable metabolites, nitrogenous compounds, and loss of freshness, particularly under conditions of inadequate handling and storage. Minimization and mitigation of environmental pollution might arise from endogenous and exogenous enzymes in the fish processing industry. Production of various fish products with industrial applications might be derived from the valorization of fish waste and discards [85]. Additionally, the concentrations of enzymes, as well as the pH and temperature, are dependent on the specific type of enzyme employed. Reported enzyme concentrations typically range from 0.01% to 5% (*w*/*w*), while the pH can vary within a range of 1.5 to 11, depending on the enzymatic activity and substrate requirements [86].

Dinakarkumar et al. [87] conducted an extraction of fish protein hydrolysate from *Secutor insidiator* using papain and proteinase K enzymes using one-variable-at-a-time optimization. The degree of hydrolysis was found to be 0.8% and 0.9% for proteinase and papain, respectively.

Recovery of protein from shrimp *Litopenaeus vannamei* waste using an aqueous two-phase system was optimized by employing CCD-coupled RSM, Genetic algorithm, and particle swarm methods. Optimal (using an RSM coupled with the Genetic algorithm) extraction parameters for protein recovery (94.99%) were achieved with a polyethylene glycol concentration of 15.8% (*w*/*w*), trisodium citrate concentration of 16.0% (*w*/*w*), pH 8.0, and temperature of 35.0 °C [41].

Many proteins and peptides are produced using chemical hydrolysis. These involve the utilization of chemical agents (such as acids or alkalis) under extreme conditions (including high temperature and/or pressure) to break the bonds between amino groups in the protein sequence. Acid hydrolysis is more prevalent in the marine industry compared to alkaline hydrolysis [84]. Chemically hydrolyzed proteins offer several advantages, including simplicity and cost-effectiveness. However, controlling the process proves challenges, resulting in protein hydrolysates of inferior nutritional and functional qualities. This can be attributed to the harsh, nonspecific cleavage of peptide bonds and the partial or complete degradation of valuable amino acids like cysteine, serine, and threonine. Alkaline hydrolysis may further lead to the formation of potentially toxic substances such as lysinoalanine, ornithinoalanine, and lanthionine [88]. Protein hydrolysate was also prepared from scallop (*Argopecten purpuratus*) byproducts using BBD-optimized enzymatic hydrolysis. At the optimum temperature (57 °C), time (62 min) and enzyme concentration (enzyme/substrate level) (0.38 Alcalase (AU)/g protein), enzymatic hydrolysis produced a hydrolysate yield of 93.92%, degree of hydrolysis of 20.44%, and protein solubility of 69.6% [58]. Moreover, protein hydrolysate production using proteolytic digestion from Monkfish (*Lophius piscatorius*) heads and viscera was optimized using the non-linear least-squares (quasi-Newton) method. About 90% of the yield of digestion was achieved at a pH of 8.3, temperature of 57.4 °C, and protease concentration of [Alcalase] = 0.05% (*v*/*w*) [37]. Moreover, protein hydrolysates production from *Scyliorhinus canicula* discards, salmonid (rainbow trout and salmon) heads, trimmings, and frames using enzymatic hydrolysis were optimized employing the non-linear least-squares (quasi-Newton) method, considering temperature, pH, enzyme concentration, ratio (solid–liquid), time of hydrolysis, and agitation speed as basic variables [38,89]. Enzymatic hydrolysis of protein hydrolysates from undersized hakes (fish bycatch) was optimized using BBD, considering parameters such as time = 2 h, solids = 50%, and enzyme/substrate = 2% [35].

### 3.3. Enzymes

Secondary raw materials derived from seafood processing encompass enzymes sourced from various parts such as the gut, liver, head, shell, and visceral organs, serving as valuable processing aids in the food industry to enhance functional and nutritive qualities [90]. These enzymes are purified using different analytical methods like ion-exchange chromatography. Saranya et al. [91] isolated an alkaline protease from fish processing waste using a combination of methods including ammonium sulfate fractionation, ion-exchange chromatography on Sephadex G-25, and DEAE column chromatography. These purification steps resulted in a four-fold increase in the purity of the protease, with a yield of 7.7%. SDS-PAGE analysis determined the molecular weight of the purified protease and estimated it to be equal to 33 kDa. The one-variable-at-a-time optimized temperature for enzyme activity was found to be 30 °C at pH 8.

Murthy et al. [92] sourced visceral proteases from little tuna (*Euthynnus affinis*), catla (*Catla catla*), and tilapia (*Oreochromis mossambicus*) originating from different habitats and isolated and characterized them using acetone, ethanol, and ammonium sulfate fractionation precipitation methods. Proteases obtained from little tuna and tilapia displayed enhanced specific activity when precipitated at 40% saturation during ammonium sulfate fractionation, with specific activities of 18.19 and 13.67 U/mg, respectively. Conversely, catla-derived enzymes exhibited the highest specific activity of 8.32 U/mg when precipitated at 60% saturation during ammonium sulfate fractionation. Acetone precipitation demonstrated superior recovery for all crude enzymes analyzed in this study. These visceral protease extraction parameters (concentration of papain (% of substrate) and concentration of crude enzyme extract of tuna viscera (% of substrate)) were optimized using D-optimal response surface design coupled with RSMs.

The chitinase-derived *Achromobacter xylosoxidans*, which was isolated from shrimp waste, exhibited full activity at an optimal temperature of 45 °C, withstanding temperatures up to 55 °C, and a pH of 8, demonstrating 80% stability [93]. The culture condition was optimized for maximum chitinase production recording up to 467 U/mL by employing Placket–Burman and central composite design statistical model.

A digestive chitosanase sourced from blue crab (*Portunus segnis*) viscera was isolated, characterized, and applied. The crude chitosanase displayed peak activity at a pH of 4.0 and a temperature of 60 °C. Moreover, it retained over 80% of its activity across a pH range spanning from 3.0 to 10.0 [94]. The chitosan hydrolysis conditions were optimized using one-variable-at-a-time, considering the most important variables such as the enzyme/substrate ratio (100 U/g) and incubation time (24 h).

The production of chitosanase was performed via the fermentation of *Paenibacillus* sp. TKU047 on squid pen waste powder. The effects of fermentation variables on the maximum production of chitosanase were optimized using one variable per time method. The maximum chitosanase production occurred when utilizing a medium containing 2% (*w*/*v*) squid pen waste powder as the sole carbon and nitrogen (C/N) source, resulting in a yield of 0.60 U/mL. The chitosanase exhibited its highest activity at a temperature of 60 °C and pH of 7. Furthermore, it demonstrated enhanced activity towards chitosan solutions with higher degrees of deacetylation (DDA) values. Additionally, the hydrolysis products obtained from 98% DDA chitosan, catalyzed using TKU047 chitosanase, revealed a degree of polymerization (DP) ranging from 2 to 9, indicating endo-type activity for the chitosanase [95]. Fermentation parameters, such as the fermentation period (24 h), MgSO_4_ (0.015%), and KCl (3%), were optimized by employing BBD for the production of chitosanase (39.774 U/g dry substrate) from marine shrimp processing raw byproducts [28]. Moreover, chitinase was produced from shrimp shell waste at optimized fermentation parameters of colloidal chitin, glucose, Tween 80 (common surfactant micelles), and yeast extract by employing CCD-coupled particle swarm optimization algorithm and artificial neural network optimization methods. The maximum chitinase activity was achieved using particle swarm optimization (115.8 U/L) and artificial neural network (124.78 U/L) optimizations. The optimum variables reported were for particle swarm optimization (colloidal chitin, 1.27%; glucose, 2.129%; Tween 80, 0.04%; yeast extract, 3.46%) and artificial neural networks (colloidal chitin, 1.4; glucose, 2.35; Tween 80, 0.07; yeast extract, 4.72) [40].

### 3.4. Carotenoids: Astaxanthins

The extraction of astaxanthin from pink shrimp waste (*Farfantepenaeus subtilis*) was carried out using palm olein at three different temperatures (50, 60, and 70 °C), optimized using a one-variable-at-a-time method [96]. Under these conditions, the maximum extraction of astaxanthin reached 29.8 µg/g of dried waste. The extraction kinetics were modeled using a simplified mass transfer kinetic model, demonstrating a strong agreement (0.969 < R^2^ < 0.991) between the experimental and calculated data.

Ultrasound-assisted natural deep eutectic solvent extraction of astaxanthin from shrimp (*Penaeus monodon*) shells was optimized by employing an RSM coupled with BBD, considering the natural deep eutectic solvent molar ratio, ultrasound amplitude, and extraction time as basic parameters. In this study, it is reported that about 68.98 mg ASX/g of shrimp waste astaxanthin was produced at an optimum natural deep eutectic solvents molar ratio = 1:1.022 (CC/LA), ultrasound amplitude = 54.43%, and extraction time = 39.23 min [57].

Liu et al. [97] carried out a solvent extraction method using dichloromethane: methanol (1:3, *v*/*v*) on shrimps and prawns (head, shell, and tail) and presented an astaxanthin content that varied from 19.2 to 7.1 µg/g. They employed one-variable-at-a-time optimization to study the extraction condition.

Hu et al. [98] employed the orthogonal test method of optimization. The mentioned optimal experimental conditions, including a solid–liquid ratio of 1:7, an extraction time of 20 min, and a temperature of 50 °C, resulted in the highest extraction yield of astaxanthin. Thus, the analysis revealed that the astaxanthin content in the *Procambarus clarkia* shell was measured at 239.96 μg/g.

Li et al. [99] reported on the high-pressure extraction of astaxanthin from shrimp byproducts, optimized using univariate analysis. Solvents’ (such as ethanol, acetone, and dichloromethane) solvation properties and pressure levels (ranging from 0 to 600 Mpa) were found to significantly influence astaxanthin extraction. High pressure was observed to disrupt cellular membranes and alter fiber structures, facilitating solvent diffusion and improving astaxanthin extraction. However, pressures exceeding 300 Mpa had a detrimental effect on astaxanthin recovery.

Ultrasound application (using parameters like 23.6% amplitude and 26.3 °C for 13.9 min) was found to enhance astaxanthin extraction from shrimp shells by employing BBD-coupled RSM optimization [34]. Fragmentation of the shell matrix was the result of cavitation induced using ultrasound, leading to increased solubility of bioactive compounds and their extraction via solvents. Solvent polarity and extraction time were identified as significant factors affecting astaxanthin yield.

An effective technique for astaxanthin extraction from crustacean byproducts was supercritical fluid extraction with the use of different solvents. RSM-coupled CCD optimized conditions (including 56.88 °C, 215.68 bar pressure, and a flow rate of solvent of 1.89 mL/min) yielded both free (12.20 µg/g) and conjugated (58.50 µg/g) astaxanthin [100]. Temperature and pressure affected the solubility of the solute in the supercritical fluid, while extraction efficiency was greatly affected by solvent selection. Higher concentrations of ethanol (5%, 10%, and 15%) led to a significant increase in astaxanthin yield (from 26.0 to 34.8 µg/g) [101]. However, astaxanthin extraction could be hindered by the application of high pressures (>400 bar) in supercritical fluid extraction optimized by using univariate analysis.

Recently, microbial fermentation followed by supercritical extraction from shrimp waste liquid fraction was optimized [102]. This BBD method’s optimized supercritical CO_2_ extraction parameters were as follows: pressure of 300 bar, temperature of 60 °C, and flow rate of 6 mL/min About 11.17% astaxanthin extraction yield from fermented shrimp waste was predicted at theses optimum variables. In this study, fermentation of the raw material by lactic acid bacteria was found to enhance astaxanthin extraction compared to common supercritical extraction methods. The extraction of lipophilic compounds in the liquor and enzymolysis of shrimp shells were increased by this fermentation, resulting in a 3.7-fold higher astaxanthin concentration (134.20 µg/g) [103].

**Figure 4:**
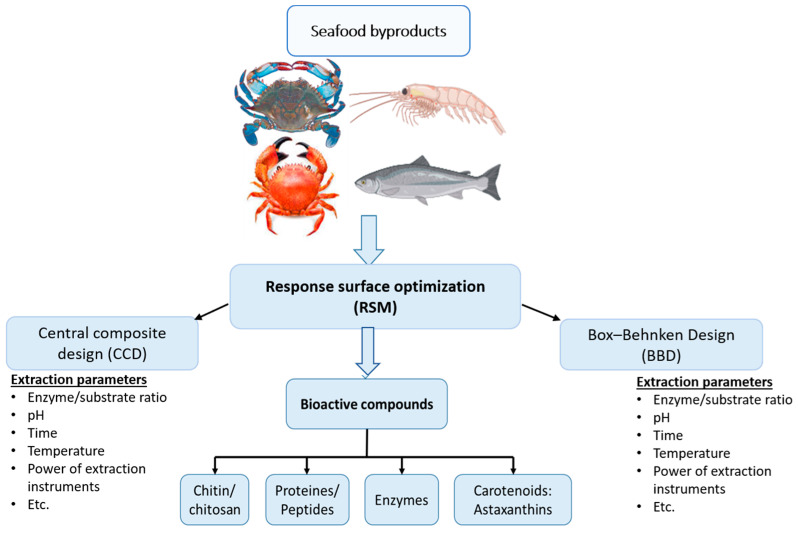
Optimization of extraction parameters of bioactive compounds from seafood byproducts.

Gulzar and Benjakul [104] investigated the combined effects of ultrasound- and pulsed-electric-field-assisted treatment on astaxanthin extraction from shrimp byproducts. The extraction yield of lipids was optimized using univariate analysis at different electric field strengths (4, 8, 12, and 16 kV cm^−1^) and pulse numbers (120, 160, 200, and 240) and an ultrasound amplitude of 80% for 25 min in continuous mode. The application of pulsed electric field pretreatment helped to reduce lipid oxidation for ultrasound-assisted extraction. They observed that disintegration, particularly in the cephalothorax, increased with higher electric field strengths. Additionally, ultrasound-induced electroporation enhances mass transfer and, consequently, improves astaxanthin recovery. Figure 4 summarizes the optimizing extraction parameters of some major seafood byproducts.

## 4. Statistical Optimization Methods Considering Economic and Quality Extraction Parameters of Bioactives

For economic and quality product development, all the innovative methods of bioactive extraction employing biological, physical, mechanical, microbial, and enzymatic processes require optimum conditions such as the concentration of solvent (solvent to substrate ratio), temperature, time, power of microwave or ultrasound, etc. In this subheading, studies focused on efficiency, quality, and processing cost optimization strategies for the extraction of bioactive substances from seafood byproducts were considered. These optimization studies were conducted to choose the best extraction technologies, check the efficiency of processing technology, select the best green extraction solvent, and/or situate the extraction processing conditions that minimize cost and maximize quality and extraction yield. Bioactive compound extraction methods are dependent on the types of the sample matrix; solvent used; and how the extraction method directly or indirectly alters the biomass properties, physical–chemical properties of the intended molecules, and their perspective end use [105]. Hence, optimizing the extraction condition that predicts and confirms the interactive effect of the dominating factors is crucial.

Optimization strategies employing RSMs coupled with CCD, Box–Behnken design, and factorial designs focused on quality, cost, and efficiency were mostly utilized for situating extraction conditions of bioactive compounds from seafood byproducts (Table 4). In particular, RSM-coupled Box–Behnken design was chosen for optimizing the improvement of extraction conditions (temperature, concentration of alkaline, time of reaction, power of microwave, and irradiation time) of chitin production from Persian Gulf shrimp waste [56]. This optimization strategy helped to differentiate the microwave-assisted extraction method from the chemical (alkaline) technique for chitosan preparation. This method was selected due to its efficiency and reduced processing costs and time. Extraction of carotenoid astaxanthin from shrimp (*Parapenaeus longirostris*) heads, thoraxes, and appendixes using supercritical fluid extraction (CO_2_ based) was proposed as an appropriate method that created quality extract (attractive antioxidant activity, pro-apoptotic, and anti-cancerous effects) and avoided the use of organic solvents for extraction [75]. Similarly, an optimized method for extracting fish lipids using microwave-assisted extraction was studied by Costa and Bragagnolo [106]. This optimized extraction method was fast and efficient and able to produce the fish lipids with acceptable fatty acid composition and no lipid oxidation. Employing high-energy extraction methods such as ultrasound-assisted extraction and microwave-assisted extraction is effective in recovering high-added-value bioactive compounds from the natural sample matrix. Optimized process conditions of these methods are faster, have low processing costs, are reproducible, and are repeatable. Optimization of these methods for the extraction of carotenoids from Red shrimp (*A. antennatus*) heads was suggested as economical and efficient [47].

Green solvent extraction methods are more cost-effective, which improves quality and enhances the recovery of oil. Moreover, ionic liquids and deep eutectic solvents have attractive biocompatibility with particular selectivity on individual bioactive compounds during extraction. This property demands optimization in conjunction with other physical parameters. Wet rendering oil recovery from catfish heads was optimized using a two-factor Taguchi orthogonal array design, considering extraction temperature and time for a better oil recovery rate. This optimization strategy was proposed to both enhance the oil extraction process and improve cost-effective fish byproduct management [32].

Most of the reactor scales for the production of bioactive compounds using enzymatic hydrolysis and fermentation methods are performed at lower volumes, thereby the optimization of the process makes it economical and easy way. These optimized processes are validated at the enlarged portion. For example, Vázquez et al. [39] studied the optimization of the protein hydrolysate production from salmonid (rainbow trout and salmon) heads, trimmings, and frames using a 100 mL reactor; then, they validated the process using a 5 L reactor. Reducing enzyme concentrations during extraction is one economic case that requires optimized utilization. For instance, during the production of salmon oil from Atlantic salmon byproducts, increasing the 50% enzyme concentration could facilitate the rate of oil recovery only by 5%, which is not economically feasible. Hence, optimizing the enzyme concentration is critical [54]. Similarly, Iñarra et al. [35] optimized protein hydrolysate extraction conditions (enzyme/substrate (protein) ratio, % solids, and time) from undersized hakes (fish bycatch) using RSM-coupled BBD that focused on developing a scaled-up model. They reported the most favorable conditions to confirm the laboratory scale at a 0.5 L and proposed a scaled-up model of 150 L concerning the protein extraction yield. One-variable-at-a-time optimization was employed to select the best bacterial isolates from seventy bacterial varieties that produce proteolytic enzymes [27]. Then, the optimal chitin extraction conditions (best bacterial isolate, carbon source, shrimp waste concentration, and inoculum size and fermentation time) were conducted using BBD-coupled RSM optimization. This optimization method increased extraction efficiency by 1.3-fold.

## 5. Optimizations on Emerging Green Extraction Technologies That Favor the Production of Potential Bioactives

In consideration of extraction variables and novel designs/instruments, this study aimed to optimize extraction process conditions before employing them in production. This optimization stage saves processing costs and time and helps to predict quality production when applied at a larger scale.

Statistical experimental designs are very critical to establishing optimized extraction processes, hydrolysis, and fermentation media conditions for the production of desired bioactive compounds from seafood byproducts. Multivariate statistical optimization methods such as RSM, artificial neural network, and non-linear least squares (quasi-Newton) coupled with different experimental designs are applicable for efficiently evaluating multiple variables that have been applied to seafood byproduct valorization. Applications of these methods for extracting bioactive compounds from different seafood byproducts are summarized in Table 4.

The chemical-treatment-based extraction (using non-polar solvents) of bioactive compounds is less acceptable due to its side effects like toxicity, environmental problems, as well as the high consumption of energy. Modern extraction methods, which involve membrane breaking or cell disruption technologies such as ultrasound- and microwave-assisted extraction, freezing/thawing, pulsed electric field, sub- and supercritical fluid extraction, and high-pressure homogenization, are more applicable to extract bioactive compounds from different sample matrixes [107]. Other green extraction technologies such as probiotic-based fermentation, enzymatic hydrolysis, and proteolytic digestion have recently been deemed acceptable for the extraction of bioactive compounds from seafood byproducts, which can solve the above-mentioned effects of organic-solvent-based treatments [27,35,37].

### 5.1. Green Solvent Extraction Parameters Optimization

Green solvents are considered to be solvents that avoid the said effects on the final product and prevent wastage. These are classified into five core groups: (1) solvents with aqueous systems; (2) ionic liquids; (3) deep eutectic solvents; (4) bio-based solvents; (5) switchable solvent systems [108]. Applying greener solvents for the extraction of bioactive substances is acceptable since they are low cost, biodegradable, non-toxic, recyclable, and safe for food- and drug-based bioactive compounds. These are grouped into neoteric solvents (ionic liquids and deep eutectic solvent), supercritical fluids (supercritical water and supercritical carbon dioxide), bio-based solvents (terpenes, glycerol, ethanol, ethyl lactate, D-limonene, etc.), and supramolecular solvents [109,110]. Choline chloridemalonic acid, a type of deep eutectic solvent, is an effective green solvent utilized for chitin extraction from shrimp shells (*Marsupenaeus japonicas*) [111]. The application protocols of using these solvents and their interaction with other extraction parameters like time, sample matrix, and temperature should be optimized for improved quality and better extraction yields. Selecting and optimizing green solvents that are suitable for the ultrasonication process is also very important. In an astaxanthin extraction from shrimp (green tiger, *Penaeus semisulcatus*) shells, suitable solvents for the ultrasonic method were initially screened and the best solvent mixtures (higher polarity) were used for optimizing the extraction conditions (ratio of solvents, extraction temperature, extraction time, and ultrasound amplitude) of astaxanthin [34].

Enzymatic processing and bacterial fermentations have been used for the production of bioactive metabolites (gelatinous solutions, oils, and protein hydrolysates) from skins and heads from megrim, hake, boarfish, grenadier, and Atlantic horse mackerel [89]. El-Bialy and Abd El-Khalek [112] studied the extraction of astaxanthin from shrimp wastes by applying two green technologies—namely, lactic fermentation and edible oil extraction. In their investigation, they found that solid-state fermentation by *Lactobacillus acidophilus* and submerged fermentation by *Streptococcus thermophilus* produced the most efficient extraction yields of astaxanthin, compared to vegetable oil (corn, flaxseed, and sesame oils)-based solvent extraction. However, vegetable-oil-based-solvent-extracted astaxanthin has shown improved medical properties such as extending shelf life and preventing microbial contamination. In developing the extraction model, parameters such as the carbon sources, type of green solvent, and fermentation time were considered. Optimizing the activity of enzymes for better extraction of bioactive compounds such as chitosanase from shrimp processing byproducts is another method to determine the quality of the product and process [28]. Optimizing consecutive extraction processes for efficient and quality bioactive production is another strategy. For instance, Vázquez et al. [37] studied two-step proteolytic digestion for the extraction of protein hydrolysates. In the first step, they optimized the hydrolysis considering the ratio of monkfish heads to water, temperature, protease concentration, and pH as basic independent variables. Then, they validated these optimum parameters for the hydrolysis of proteins from the head and viscera of monkfish. Creating optimum enzymatic hydrolysis conditions (temperature and pH) to produce protein hydrolysates from *Scyliorhinus canicula* discards by employing the non-linear least-squares (quasi-Newton) method was studied by Vázquez et al. [38]. Fish skins were studied as an excellent and easily available resource for collagen extraction. This extraction processes was optimized in a two-step process by Blanco et al. [45]. First, they optimized the extractability of collagen (extraction yield) from Small-Spotted Catshark (*S. canicula*) skin, considering NaOH concentration, time, and temperature. Then, the optimum conditions were used to optimize the yield and aminioacid quality using acetic acid concentration, temperature, and time as independent factors. Moreover, Box–Behnken design coupled with RSM optimization was employed for the deproteinization process of chitin extraction conditions (pH, time, temperature, agitation speed, and enzyme-to-substrate ratio) [63]. The production of protein hydrolysate from scallop (*Argopecten purpuratus*) visceral meal and defatted meal with enhanced proximal composition, amino acid composition, yield, molecular profile, protein solubility, and degree of hydrolysis was optimized using an RSM coupled with BBD. Three basic independent variables (temperature, time, and enzyme concentration (enzyme/substrate level)) were optimized [58].

### 5.2. Optimizing Physical Processing (Cell Wall Breakdown) Extraction Parameters

The applications of ultrasound-assisted extraction of bioactive compounds from seafood sample matrices are mainly affected by factors such as high temperatures and pressures. A pressurized area is created on the bubbled solvent, which then fiercely discharges the liquid part from the sample cells. The interactive effect of other factors such as ultrasonic frequency, intensity, and processing time affect the extraction capacity, based on the above factors [7]. Hence, statistical optimizations that optimize the suitable extraction conditions for better efficiency, quality, and lower processing costs and time are required. Protein extraction optimization requires consideration of extraction parameters and technology that facilitate the cell wall breakdown without side effects on the final product. Unless a suitable and optimized extraction method is developed, fish protein is highly sensitive and can be degraded via uncontrolled extraction factors like oxidation and denaturation via excessive heat.

**Figure 5:**
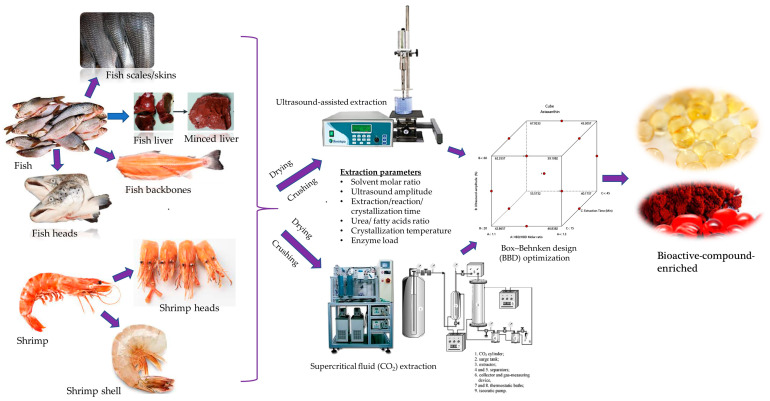
Typical optimization of bioactive compound extraction from seafood byproducts using green extraction technologies [55,115].

RSM-coupled BBD was employed to differentiate the efficiency of ultrasound-assisted extraction and microwave-assisted extraction of carotenoids from Red shrimp (*A. antennatus*) heads. In this study, the extraction time, ultrasound, microwave power, and solvent/material ratio were considered as independent variables. This ultrasound-assisted extraction was efficient, had lower processing time, and a lower solvent/material ratio than the microwave-assisted extraction [47]. Microwave-assisted extraction of chitosan from Persian Gulf shrimp (species of *P. indicus*) under optimized extraction parameters of temperature, NaOH concentration, power of irradiation, and time of reaction was more effective than the chemical (alkaline) method [56]. Bioactive fish oil extraction was optimized as a sustainable method for valorizing fish byproducts (heads and fins) using microwave-assisted extraction. The independent variables of the extraction process considered to be optimized for obtaining high quality and oil yield were time, microwave power, and the solid–liquid ratio. The optimum microwave-assisted extraction recovered from 60% to 100% of oil at about 19 min and with less solvent utilization compared to Soxhlet extraction [3]. A typical optimization of bioactive compounds extraction from fish and shrimp byproducts using green extraction technologies is depicted in Figure 5.

Manothermosonication is a type of ultrasonic extraction which works by combining pressure, temperature and ultrasound intensity to facilitate the extraction of water-soluble bioactive compounds from a sample matrix. This is because the method not only facilitates cell disruption but also enhances mass transfer phenomena or effective diffusivity for better extraction yield [113]. Thus, assuring the optimum interactive effect of these extraction parameters is very important.

A study was conducted to compare conventional hexane pressing extraction methods and supercritical carbon dioxide extraction methods of cod liver oil from cod fish visceral parts. The supercritical carbon dioxide extraction method was optimized considering temperature, pressure, and CO_2_ flow rate. This RSM-optimized SC-CO_2_ extraction method was highlighted as producing the most efficient and high quality liver oil (best antioxidant and anticancer activities; highest squalene, vitamin D_3_, and vitamin K content), compared to other methods [80]. The combined effects of subcritical dimethyl ether extraction parameters of oil from high-moisture tuna liver were optimized using the ratio of temperature to pressure, time, and stirring speed by employing RSM. At optimum extraction conditions of this method, the oil extraction yield was comparable to supercritical carbon dioxide extraction of tuna liver oil [114].

## 6. Conclusions

Selecting the best statistical optimization strategies to optimize the extraction conditions of bioactive compounds from seafood byproducts using conventional and green technologies is an inevitable research activity. In this review, RSM-coupled CCD and BBD have been shown to be the most-employed optimizing strategies of bioactive compound extraction parameters. The dominant extraction parameters considered for optimizations were the enzyme/substrate ratio, pH, time, temperature, and power of extraction instruments. The effects of these independent variables on the extraction capacities and qualities of the bioactive compounds, chitin and chitosan, proteins and peptides, and enzymes and carotenoids (astaxanthins) were optimized using the above optimization methods. Most of the studies have shown limitations in indicating if confirmation experiments at those developed optimum points were conducted for validation of their developed optimization model.

## Figures and Tables

**Table 1:** Some RSM equations used to depict total percentage contributions (TPCs) of extraction variables for bioactive compound extraction responses.

DoE	Developed Equation	Number of Factors	*p*	Percentage Contribution of Variables (%)			Reference
				TPC*_i_*	TPC*_ii_*	TPC*_ij_*	
CCD	*Y* = 39.2 + 9.3*X*_1_ + 3.1*X*_2_ + 4.1*X*_3_ − 3.4*X*^2^_3_	3	5	86.5	13.5		[45]
	*Y* = 82.46 − 2.43*X*_1_ + 5.23*X*_2_ + 7.02*X*_3_ + 0.64*X*_4_ + 0.31*X*_1_*X*_2_ + 0.35*X*_1_*X*_3_ + 0.33*X*_1_*X*_4_ − 0.16*X*_2_*X*_3_ + 0.1*X*_2_*X*_4_ − 0.5*X*_3_*X*_4_ − 10.6*X*^2^_1_ + 0.4*X*^2^_2_ − 0.051*X*^2^_3_ + 16.62*X*^2^_4_	4	15	59.5	40.1	0.4	[41]
	*Y* = −120.3 + 416*X*_1_ + 2.8*X*_2_ + 9.2*X*_3_ − 3.6*X*^2^_1_ − 0.01*X*^2^_2_ − 0.2*X*^2^_3_ − 0.14*X*_1_*X*_2_ − 0.8*X*_1_*X*_3_ − 0.04*X*_2_*X*_3_	3	10	80.8	16.2	3.0	[26]
	*Y* = 14.4 + 0.8*X*_1_ + 0.04*X*_2_ − 1.5*X*_3_ − 0.45*X*^2^_3_ − 0.3*X*_1_*X*_3_ + 0.4*X*_2_*X*_3_ *Y* = 19.5 + 0.52*X*_1_ + 1.2*X*_2_ − 1*X*_3_ − 1.25*X*^2^_1_ − 1*X*^2^_2_ − 0.3*X*_2_*X*_3_	3 3	7 7	88.7 51.2	6.8 47.6	4.5 1.2	[3]
BBD	*Y* = 39.2 + 21.2*X*_1_ − 3.7*X*_2_ − 0.066*X*_3_ + 0.154*X*_1_*X*_2_ + 0.045*X*_1_*X*_3_ + 0.003 *X*_2_*X*_3_ − 0.64*X*^2^_1_ − 0.1*X*^2^_2_ + 0.004*X*^2^_3_	3	10	86.2	13.5	0.3	[59]
	*Y* = −33.1 + 0.81*X*_1_ + 0.6*X*_2_ + 85.3*X*_3_ − 0.008*X*^2^_1_ − 0.003*X*^2^_2_ − 91.2*X*^2^_3_ − 0.003*X*_1_*X*_2_ − 0.3*X*_1_*X*_3_	3	9	51.1	44.3	4.6	[58]
	*Y* = −9.9 + 11.5*X*_1_ + 1.7*X*_2_ + 1.7*X*_3_ − 0.09*X*_1_*X*_2_ − 0.32*X*_1_*X*_3_ − 0.006*X*_2_*X*_3_ − 0.7*X*^2^_1_ − 0.01*X*^2^_2_ − 0.1*X*^2^_3_	3	10	64.2	19.7	16.1	[57]
	*Y* = 4.9 + 0.9*X*_1_ + 0.5*X*_2_ − 0.4*X*_3_ − 0.3*X*^2^_1_ − 1*X*^2^_2_ − 0.4*X*^2^_3_ *Y* = 7.1 + 0.9*X*_1_ + 0.5*X*_2_ − 0.4*X*_3_ − 0.5*X*^2^_1_ − 1.2*X*^2^_2_ − 0.5*X*^2^_3_	3 3	7 7	68.2 52.2	30.9 43.0	0.9 4.8	[56]
	*Y* = −18.1 + 3.2*X*_1_ − 580.2*X*_2_ + 0.02*X*_3_ − 0.05*X*^2^_1_ + 49269.7*X*^2^_2_ + 0.27*X*^2^_3_ − 16.6*X*_1_*X*_2_ + 0.13*X*_1_*X*_3_ − 47.5*X*_2_*X*_3_	3	10	72.9	26.2	0.9	[28]
	*Y* = 63.7 − 63.7*X*_1_ − 5.8*X*_2_ − 3*X*_3_ + 16.6*X*_4_ + 5.8*X*_1_*X*_2_ + 6.14*X*_1_*X*_3_ − 2.9*X*_1_*X*_4_ − 0.24*X*_2_*X*_4_ − 0.3*X*_3_*X*_4_ − 1.4*X*^2^_1_ − 4.7*X*^2^_2_ − 4.34*X*^2^_3_ + 1.8*X*^2^_4_	4	14	86.2	6.6	7.2	[27]
	*Y* = 10.7 + 1.3*X*_1_ + 0.1*X*_2_ + 2.2*X*_3_ + 0.4*X*^2^_1_ − 0.6*X*^2^_2_ + 1*X*^2^_3_ − 0.8*X*_1_*X*_2_ + 0.25*X*_1_*X*^2^_2_ + 0.8*X*^2^_1_*X*_2_ + 0.7*X*_1_*X*_3_ − 0.3*X*^2^_1_*X*_3_ + 0.4*X*_2_*X*_3_ *Y* = 15.9 + 0.6*X*_1_ + 0.5*X*_2_ + 0.7*X*_3_ − 0.9*X*^2^_1_ − 0.4*X*^2^_2_ + 0.4*X*^2^_3_ + 0.23*X*_1_*X*_2_ − 1.55*X*_1_*X*^2^_2_ + 0.5*X*^2^_1_*X*_2_ + 1*X*_1_*X*_3_ + 1*X*^2^_1_*X*_3_ − 0.6*X*_2_*X*_3_	3 3	13 13	18.1 30.6	68.0 14.0	13.9 55.4	[47]
Full Factorial Design	1. *Y* = 528.9 − 29.04*X*_1_ + 0.87*X*^2^_1_ − 164.8*X*_3_ + 23.2*X*^2^_3_ 2. *Y* = 28.8 − 0.0013*X*^2^_1_ − 0.1*X*_2_ − 12.7*X*_3_ + 1.8*X*^2^_3_ 3. *Y* = 121.1 − 78.4*X*_1_ + 49.3*X*^2^_1_ − 44.2*X*_3_ + 31.9*X*^2^_3_	3	5 5 5	98.1 98.0 70.1	0.003 0.006 21.0	1.9 2.0 8.8	[60]
	Y = − 722.4 + 1.6*X*_1_ + 28.3*X*_2_ − 0.6*X*_3_ + 83*X*_4_ + 0.002*X*^2^_1_ − 0.3*X*^2^_2_ − 0.11*X*^2^_3_ − 9.5*X*^2^_4_ + 0.004*X*_1_*X*_2_ − 0.05*X*_1_*X*_3_ − 0.035*X*_1_*X*_4_ + 0.1*X*_2_*X*_3_ − 0.2*X*_2_*X*_4_ + 0.3*X*_3_*X*_4_	4	15	10.3	1.2	88.5	[54]

*p* = number of coefficients of the developed model. *Y* = dependent variable/response/yield of the focused bioactive compound extracted from seafood byproduct. *X*_1_, *X*_2_, *X*_3_, and *X*_4_ = optimized independent variable/factors/parameters that influence/affect the dependent variable/response/yield. TPC*_i_* is the total percentage contributions (%) of linear terms, TPC*_ii_* is the total percentage contributions (%) of quadratic terms, and TPC*_ij_* is the interaction terms.

**Table 2:** Model adequacy evaluation of statistical parameters for a developed model to predict the extraction of astaxanthin yield from shrimp shells.

Statistical Parameter	Value
Std. Dev.	1.19
C.V. %	2.09
R^2^	0.9870
Adjusted R^2^	0.9702
Predicted R^2^	0.8990
Adeq Precision	24.6656
PRESS	77.19
AAD (%)	1.07

Std. Dev. = standard deviation, C.V. = coefficient of variance, PRESS = predicted error sum of square, and AAD = absolute average deviation.

**Table 3:** Classical (one-variable-at-a-time) methods of statistical optimization strategies applied for the extraction of bioactive molecules from seafood byproducts.

Seafood Byproduct Type	Design Method of Experiments (DoE)	Employed Software	Extraction Method	Targeted Bioactive Molecule	Considered Extraction Parameter/s	Reference
Red Shrimp (*Aristeus alcocki*) shell waste	Analysis of variance technique	SPSS 15	Non-deproteinization of enzymatic digestion	Carotenoids	Different organic solventsThree different vegetable oils	[67]
Fish scales and feather wastes	Analysis of variance technique		*Bacillus* sp. CL18 as a bioconverter	Protease; bioactive hydrolysates	Twelve substrates and co-substrates	[68]
Sea bass skinhead, tail, thorns, and backbone	Analysis of variance technique	InfoStatfi and StatAdvisorfi version 2018	Bacterial fermentation	Phenolic acids	Fermentation time (in hours)	[69]
Comb penshell (*Atrina pectinata*)	One-way analysis of variance	SPSS version 23	Subcritical water hydrolysis	Amino acids and marine bioactive peptides	Extraction temperatures	[70]
Crustacean shell waste	One-way analysis of variance	Sigma Plot 14.0	Submerged fermentation	Chitinase; protease	fermentation time, pH, and temperature	[71]
Speckled shrimp *Metapenaeus monoceros* shells	One-way analysis of variance	SPSS Version 11.0.1.2001	Flask based hydrolysis	Protease	Concentrations of shrimp; sugar	[31]
Speckled shrimp *Metapenaeus monoceros* shells	One-way analysis of variance	SPSS ver.17.0	Deproteinization of enzymatic digestion	Deproteinized bioactive hydrolysate	enzyme/substrate ratios	[72]
shrimp (*P. kerathurus*) shells and blue crabs (*P. segnis*) viscera	One-way analysis of variance	SPSS ver.17.0	Deproteinization of enzymatic digestion	Chitin	pH and temperature	[73]
Shrimp (*Parapenaeus longirostris*) heads, thorax, appendix cephalothorax and abdominal parts	One-way analysis of variance	SPSS version 20.0	Supercritical CO_2_ extraction	Astaxanthin and peptides Carotenoid astaxanthin	Extraction rate	[74,75]
Shrimp (*Penaeus merguiensis*) shells	One-way analysis of variance	SPSS version 19.0	Fermentation	Chitin; chitosan	Differences in bacterial strains	[76]
Shrimp shells powders	One-way analysis of variance	SPSS version 19.0	Submerged fermentation	Chitin	Time; dilution; 2% diethyl sulfate; UV irradiation; microwave heating treatments	[77]
Head, skins, and viscera of rainbow trout (*Oncorhynchus mykiss*) and Sole (*Dover sole*)	One-way analysis of variance	SPSS	Accelerated solvent extraction and pulsed electric fields	Protein content	Temperature, time, pH, and pressure	[78]
Blue crab (*Portunus segnis*) shells	One-way analysis of variance	SPSS ver. 17.0	Enzymatic pretreatment combined with solvent maceration	Carotenoproteins	Time intervals and concentration *Portunus segnis* proteases	[79]

**Table 4:** Multivariate techniques of statistical optimization strategies applied for the extraction of bioactive molecules from seafood byproducts.

Seafood Byproduct Type	Statistical Methodology	Design Method of Experiments (DoE)	Employed Software	Extraction Method	Targeted Bioactive Molecule	Considered Extraction Parameters	Reference
Cod fish liver	RSM			Conventional hexane and supercritical carbon dioxide	Cod liver oil	temperature, pressure, and CO_2_ flow rate	[80]
Shrimp shell waste	Particle swarm optimization algorithm and artificial neural network	CCD	MATLAB R2016a	Fermentation	Chitinase	Colloidal chitin, glucose, Tween 80 (common surfactant micelles), and yeast extract	[40]
Shrimp (*Penaeus* sp.) cephalothoraxes and carapaces	RSM	Fractional factorial design (FFD) CCD	Statsoft 1997	Thermochemical treatments	Chitin	Concentration of HCl solution, solid–liquid ratio of HCl solution, number of treatments, concentration of NaOH solution, reaction time, reaction temperature, and solid–liquid ratio of NaOH solution	[26]
Shrimp (*Litopenaeus vannamei)* waste	RSM Genetic algorithm and particle swarm	CCD	Design-Expert software (version 10.0.1.0	Aqueous two-phase system	Protein recovery	Polyethylene glycol concentration, trisodium citrate concentration, pH, and temperature	[41]
Speckled shrimp (*Metapenaeus monoceros)* shells	RSM	Taguchi’s L27; Box–Behnken Design	SPSS Version 11.0.1.2001	Flask-based hydrolysis	Chitin	Temperature, inoculum size of strain, and culture volume	[31]
Shrimp heads	RSM	3-level fractional factorial	Statistica software Version 10	Ultrasound and microwave assisted extraction	Phenolic and carotenoids	Extraction time; solvent-to-propolis and Choline Chloride: Tartaric Acid-to-H_2_O ratios	[60]
Atlantic salmon frame bone	RSM	BBD	Design-Expert v. 7 Trail	Supercritical carbon dioxide (SC-CO_2_)	Oil	Urea/ fatty acids ratio, crystallization temperature, and crystallization time	[59]
Small-Spotted Catshark (*S. canicula*) skin	RSM	CCRD	Microsoft Excel spreadsheet (version 10)	Alkaline pretreatment; acid-soluble collagen extraction	Collagen	Chemical treatment (NaOH) concentration, temperature and time, and concentration of acetic acid	[45]
Scallops (*Argopecten purpuratus*) byproducts	RSM	BBD	Minitab 19	Enzymatic Hydrolysis	Protein hydrolysate	Temperature, time, and enzyme concentration (enzyme/substrate level)	[58]
Shrimp (*Penaeus monodon*) shells	RSM	BBD	Design-Expert software (version 7.0.0)	Ultrasound-assisted natural deep eutectic solvents	Astaxanthin	Natural deep eutectic solvents molar ratio, ultrasound-amplitude, and extraction time	[57]
Indian white shrimp waste	RSM	BBD	Design Expert 7.1.6 and Minitab 16 statistical software	Chemical and microwave method	Chitosan	Temperature, concentration of alkaline, time of reaction, power of microwave, and irradiation time	[56]
Marine shrimp processing raw byproducts		Plackett–Burman and BBD		Fermentation	Chitosanase	Fermentation period, temperature, period of microwave pretreatment, K_2_HPO_4_ (%), MgSO_4_ (%), KCl (%), and FeSO_4_·7H2O	[28]
Salmon (*Salmo salar*) backbones, heads, and viscera	RSM	Central composite rotatable design	Design-Expert Version 11	Soxhlet and microwave-assisted extraction	Bioactive oils	Time, microwave power, and solid–liquid ratio	[53]
Monkfish (*Lophius piscatorius*) heads and viscera	Non-linear least-squares (quasi-Newton) method	Data-fitting and parametric estimations	Solver of Excel spreadsheet	Proteolytic digestion	Protein hydrolysates	pH, temperature, and protease concentration	[37]
Shrimp (*Parapenaeus longirostris*) shells waste	RSM	BBD	STATISTICA	Fermentation	Chitin and chitosan	Sucrose concentration, shrimp shell waste concentration, inoculum size, and incubation period	[27]
Undersized hakes (fish bycatch)	RSM	Box–Behnken Design	Statgraphics Centurion XVI	Enzymatic Hydrolysis	Protein hydrolysates	Enzyme/substrate (protein) ratio, % solids, and time	[35]
Black tiger shrimp (*Penaeus monodon*) shells	RSM	BBD	Sigmaplot-11 Excel	Enzymatic Hydrolysis	chitin	pH, temperature, agitation speed, enzyme substrate ratio, incubation time	[63]
*Scyliorhinus canicula* discards	Non-linear least-squares (quasi-Newton) method	Rotatable second-order design	SolverAid, Microsoft Excel spreadsheet	Enzymatic Hydrolysis	Protein hydrolysates	Temperature and pH	[38]
Red shrimps, (*A. antennatus*) head	RSM	BBD	Statistica Version 10	Ultrasound-assisted, microwave-assisted extraction	Carotenoids	Extraction time, ultrasound, microwave power, and solvent/material ratio	[47]
Atlantic salmon (*Salmo salar*) heads, frames, and viscera	RSM	Factorial design	Minitab 17.1	Enzymatic transesterification	Oil; biodiesel	Enzyme concentration, oil/alcohol molar ratio, time, and temperature	[54]
Fish byproduct: heads, fins	RSM	CCRD	Design-Expert, Version 11	Microwave-assisted extraction	Bioactive fish oil	Time, microwave power, and solid–liquid ratio	[3]
Salmonids (rainbow trout and salmon) heads, trimmings, and frames	Non-linear least-squares (quasi-Newton) method	Second-order rotatable design	Solver, Microsoft Excel spreadsheet	Enzymatic Hydrolysis	Protein hydrolysates	Enzyme concentration, pH, ratio (solid:liquid, time of hydrolysis, and agitation speed	[39]

## References

[1] Kumar A., Kumar D., George N., Sharma P., Gupta N. (2018). A process for complete biodegradation of shrimp waste by a novel marine isolate *Paenibacillus* sp. AD with simultaneous production of chitinase and chitin oligosaccharides. Int. J. Biol. Macromol..

[2] Coelho T.L.S., Silva D.S.N., dos Santos Junior J.M., Dantas C., Nogueira A.R.d.A., Lopes Júnior C.A., Vieira E.C. (2022). Multivariate optimization and comparison between conventional extraction (CE) and ultrasonic-assisted extraction (UAE) of carotenoid extraction from cashew apple. Ultrason. Sonochem..

[3] Pinela J., Fuente B.d.l., Rodrigues M., Pires T.C.S.P., Mandim F., Almeida A., Dias M.I., Caleja C., Barros L. (2023). Upcycling Fish By-Products into Bioactive Fish Oil: The Suitability of Microwave-Assisted Extraction. Biomolecules.

[4] Lefebvre T., Destandau E., Lesellier E. (2021). Selective extraction of bioactive compounds from plants using recent extraction techniques: A review. J. Chromatogr. A.

[5] Gunasekaran J., Kannuchamy N., Kannaiyan S., Chakraborti R., Gudipati V. (2015). Protein Hydrolysates from Shrimp (*Metapenaeus dobsoni*) Head Waste: Optimization of Extraction Conditions by Response Surface Methodology. J. Aquat. Food Prod. Technol..

[6] Ozogul F., Cagalj M., Šimat V., Ozogul Y., Tkaczewska J., Hassoun A., Kaddour A.A., Kuley E., Rathod N.B., Phadke G.G. (2021). Recent developments in valorisation of bioactive ingredients in discard/seafood processing by-products. Trends Food Sci. Technol..

[7] Ali A., Wei S., Liu Z., Fan X., Sun Q., Xia Q., Liu S., Hao J., Deng C. (2021). Non-thermal processing technologies for the recovery of bioactive compounds from marine by-products. LWT.

[8] Martins R., Barbosa A., Advinha B., Sales H., Pontes R., Nunes J. (2023). Green Extraction Techniques of Bioactive Compounds: A State-of-the-Art Review. Processes.

[9] Picot-Allain C., Mahomoodally M.F., Ak G., Zengin G. (2021). Conventional versus green extraction techniques—A comparative perspective. Curr. Opin. Food Sci..

[10] Ojha K.S., Aznar R., O’Donnell C., Tiwari B.K. (2020). Ultrasound technology for the extraction of biologically active molecules from plant, animal and marine sources. TrAC Trends Anal. Chem..

[11] Ghalamara S., Silva S., Brazinha C., Pintado M. (2020). Valorization of Fish By-Products: Purification of Bioactive Peptides from Codfish Blood and Sardine Cooking Wastewaters by Membrane Processing. Membranes.

[12] Chemat F., Vian M.A., Cravotto G. (2012). Green Extraction of Natural Products: Concept and Principles. Int. J. Mol. Sci..

[13] Benvenutti L., Zielinski A.A.F., Ferreira S.R.S. (2019). Which is the best food emerging solvent: IL, DES or NADES?. Trends Food Sci. Technol..

[14] Saini A., Panesar P.S. (2020). Beneficiation of food processing by-products through extraction of bioactive compounds using neoteric solvents. LWT.

[15] El-Shamy S., Farag M.A. (2021). Novel trends in extraction and optimization methods of bioactives recovery from pomegranate fruit biowastes: Valorization purposes for industrial applications. Food Chem..

[16] Dayakar B., Xavier M., Ngasotter S., Dhanabalan V., Porayil L., Balange A.K., Nayak B.B. (2023). Extraction, optimization, and functional quality evaluation of carotenoproteins from shrimp processing side streams through enzymatic process. Environ. Sci. Pollut. Res..

[17] Ahmadkelayeh S., Cheema S.K., Hawboldt K. (2022). Supercritical CO_2_ extraction of lipids and astaxanthin from Atlantic shrimp by-products with static co-solvents: Process optimization and mathematical modeling studies. J. CO_2_ Util..

[18] Vanaja K., Shobha Rani R. (2007). Design of experiments: Concept and applications of Plackett Burman design. Clin. Res. Regul. Aff..

[19] Yolmeh M., Jafari S.M. (2017). Applications of response surface methodology in the food industry processes. Food Bioprocess Technol..

[20] Honrado A., Rubio S., Beltrán J.A., Calanche J. (2022). Fish by-Product Valorization as Source of Bioactive Compounds for Food Enrichment: Characterization, Suitability and Shelf Life. Foods.

[21] He C., Cao J., Bao Y., Sun Z., Liu Z., Li C. (2021). Characterization of lipid profiling in three parts (muscle, head and viscera) of tilapia (*Oreochromis niloticus*) using lipidomics with UPLC-ESI-Q-TOF-MS. Food Chem..

[22] Latha S., Sivaranjani G., Dhanasekaran D. (2017). Response surface methodology: A non-conventional statistical tool to maximize the throughput of Streptomyces species biomass and their bioactive metabolites. Crit. Rev. Microbiol..

[23] Trocine L., Malone L.C. Finding important independent variables through screening designs: A comparison of methods. Proceedings of the 2000 Winter Simulation Conference Proceedings (Cat. No. 00CH37165).

[24] Khalil M., Darusman L.K., Syafitri U.D. (2011). Application of fractional factorial design to optimize extraction method of artemisinin from *Artemisia annua*. ScienceAsia.

[25] Ye Z., Wang W., Yuan Q., Ye H., Sun Y., Zhang H., Zeng X. (2016). Box–Behnken design for extraction optimization, characterization and in vitro antioxidant activity of *Cicer arietinum* L. hull polysaccharides. Carbohydr. Polym..

[26] Nidheesh T., Suresh P.V. (2015). Optimization of conditions for isolation of high quality chitin from shrimp processing raw byproducts using response surface methodology and its characterization. J. Food Sci. Technol..

[27] Gamal R.F., El-Tayeb T.S., Raffat E.I., Ibrahim H.M.M., Bashandy A.S. (2016). Optimization of chitin yield from shrimp shell waste by *Bacillus subtilis* and impact of gamma irradiation on production of low molecular weight chitosan. Int. J. Biol. Macromol..

[28] Ismail S.A. (2019). Microbial valorization of shrimp byproducts via the production of thermostable chitosanase and antioxidant chitooligosaccharides. Biocatal. Agric. Biotechnol..

[29] Chen X., Ding J., Ji D., He S., Ma H. (2020). Optimization of ultrasonic-assisted extraction conditions for bioactive components from coffee leaves using the Taguchi design and response surface methodology. J. Food Sci..

[30] Simon S., K S., Joseph J., George D. (2023). Optimization of extraction parameters of bioactive components from *Moringa oleifera* leaves using Taguchi method. Biomass Convers. Biorefin..

[31] Jabeur F., Mechri S., Kriaa M., Gharbi I., Bejaoui N., Sadok S., Jaouadi B. (2020). Statistical Experimental Design Optimization of Microbial Proteases Production under Co-Culture Conditions for Chitin Recovery from Speckled Shrimp *Metapenaeus monoceros* by-Product. BioMed Res. Int..

[32] Dave J., Ali A.M.M., Kudre T., Nukhthamna P., Kumar N., Kieliszek M., Bavisetty S.C.B. (2023). Influence of solvent-free extraction of fish oil from catfish (*Clarias magur*) heads using a Taguchi orthogonal array design: A qualitative and quantitative approach. Open Life Sci..

[33] Gao J., You J., Kang J., Nie F., Ji H., Liu S. (2020). Recovery of astaxanthin from shrimp (*Penaeus vannamei*) waste by ultrasonic-assisted extraction using ionic liquid-in-water microemulsions. Food Chem..

[34] Sharayei P., Azarpazhooh E., Zomorodi S., Einafshar S., Ramaswamy H.S. (2021). Optimization of ultrasonic-assisted extraction of astaxanthin from green tiger (*Penaeus semisulcatus*) shrimp shell. Ultrason. Sonochem..

[35] Iñarra B., Bald C., Gutierrez M., San Martin D., Zufía J., Ibarruri J. (2023). Production of Bioactive Peptides from Hake By-Catches: Optimization and Scale-up of Enzymatic Hydrolysis Process. Mar. Drugs.

[36] Bezerra M.A., Santelli R.E., Oliveira E.P., Villar L.S., Escaleira L.A. (2008). Response surface methodology (RSM) as a tool for optimization in analytical chemistry. Talanta.

[37] Vázquez J.A., Menduíña A., Nogueira M., Durán A.I., Sanz N., Valcarcel J. (2020). Optimal Production of Protein Hydrolysates from Monkfish By-Products: Chemical Features and Associated Biological Activities. Molecules.

[38] Vázquez J.A., Blanco M., Massa A.E., Amado I.R., Pérez-Martín R.I. (2017). Production of Fish Protein Hydrolysates from *Scyliorhinus canicula* Discards with Antihypertensive and Antioxidant Activities by Enzymatic Hydrolysis and Mathematical Optimization Using Response Surface Methodology. Mar. Drugs.

[39] Vázquez J.A., Sotelo C.G., Sanz N., Pérez-Martín R.I., Rodríguez-Amado I., Valcarcel J. (2019). Valorization of Aquaculture By-Products of Salmonids to Produce Enzymatic Hydrolysates: Process Optimization, Chemical Characterization and Evaluation of Bioactives. Mar. Drugs.

[40] Suryawanshi N., Eswari J.S. (2022). Chitin from seafood waste: Particle swarm optimization and neural network study for the improved chitinase production. J. Chem. Technol. Biotechnol..

[41] Saravana Pandian P., Sindhanaiselvan S., Subathira A., Saravanan S. (2023). A correlative algorithmic optimization study for an integrated soft computing technique in aqueous two-phase protein extraction from Litopenaeus vannamei waste. Biomass Convers. Biorefin..

[42] Kennedy J., Eberhart R. Particle swarm optimization. Proceedings of the ICNN’95-International Conference on Neural Networks.

[43] Selber K., Nellen F., Steffen B., Thömmes J., Kula M.-R. (2000). Investigation of mathematical methods for efficient optimisation of aqueous two-phase extraction. J. Chromatogr. B Biomed. Sci. Appl..

[44] Ferreira S.L.C., Silva Junior M.M., Felix C.S.A., da Silva D.L.F., Santos A.S., Santos Neto J.H., de Souza C.T., Cruz Junior R.A., Souza A.S. (2019). Multivariate optimization techniques in food analysis—A review. Food Chem..

[45] Blanco M., Vázquez J.A., Pérez-Martín R.I., Sotelo C.G. (2019). Collagen Extraction Optimization from the Skin of the Small-Spotted Catshark (*S. canicula*) by Response Surface Methodology. Mar. Drugs.

[46] Srinivasan H., Kanayairam V., Ravichandran R. (2018). Chitin and chitosan preparation from shrimp shells *Penaeus monodon* and its human ovarian cancer cell line, PA-1. Int. J. Biol. Macromol..

[47] Tsiaka T., Zoumpoulakis P., Sinanoglou V.J., Makris C., Heropoulos G.A., Calokerinos A.C. (2015). Response surface methodology toward the optimization of high-energy carotenoid extraction from *Aristeus antennatus* shrimp. Anal. Chim. Acta.

[48] Paulo F., Tavares L., Santos L. (2022). Response Surface Modeling and Optimization of the Extraction of Phenolic Antioxidants from Olive Mill Pomace. Molecules.

[49] Maran J.P., Manikandan S., Thirugnanasambandham K., Nivetha C.V., Dinesh R. (2013). Box–Behnken design based statistical modeling for ultrasound-assisted extraction of corn silk polysaccharide. Carbohydr. Polym..

[50] Khataee A.R., Fathinia M., Aber S., Zarei M. (2010). Optimization of photocatalytic treatment of dye solution on supported TiO_2_ nanoparticles by central composite design: Intermediates identification. J. Hazard. Mater..

[51] Abdessalem A.K., Oturan N., Bellakhal N., Dachraoui M., Oturan M.A. (2008). Experimental design methodology applied to electro-Fenton treatment for degradation of herbicide chlortoluron. Appl. Catal. B Environ..

[52] Yetilmezsoy K., Demirel S., Vanderbei R.J. (2009). Response surface modeling of Pb (II) removal from aqueous solution by *Pistacia vera* L.: Box–Behnken experimental design. J. Hazard. Mater..

[53] de la Fuente B., Pinela J., Mandim F., Heleno S.A., Ferreira I.C., Barba F.J., Berrada H., Caleja C., Barros L. (2022). Nutritional and bioactive oils from salmon (*Salmo salar*) side streams obtained by Soxhlet and optimized microwave-assisted extraction. Food Chem..

[54] Ramakrishnan V.V., Dave D., Liu Y., Routray W., Murphy W. (2021). Statistical Optimization of Biodiesel Production from Salmon Oil via Enzymatic Transesterification: Investigation of the Effects of Various Operational Parameters. Processes.

[55] Haq M., Pendleton P., Chun B.-S. (2020). Utilization of Atlantic Salmon By-product Oil for Omega-3 Fatty Acids Rich 2-Monoacylglycerol Production: Optimization of Enzymatic Reaction Parameters. Waste Biomass Valorization.

[56] Nouri M., Khodaiyan F., Razavi S.H., Mousavi M. (2016). Improvement of chitosan production from Persian Gulf shrimp waste by response surface methodology. Food Hydrocoll..

[57] Chandra Roy V., Ho T.C., Lee H.-J., Park J.-S., Nam S.Y., Lee H., Getachew A.T., Chun B.-S. (2021). Extraction of astaxanthin using ultrasound-assisted natural deep eutectic solvents from shrimp wastes and its application in bioactive films. J. Clean. Prod..

[58] Chasquibol N., Gonzales B.F., Alarcón R., Sotelo A., Márquez-López J.C., Rodríguez-Martin N.M., del Carmen Millán-Linares M., Millán F., Pedroche J. (2023). Optimisation and Characterisation of the Protein Hydrolysate of Scallops (*Argopecten purpuratus)* Visceral By-Products. Foods.

[59] Haq M., Getachew A.T., Saravana P.S., Cho Y.-J., Park S.-K., Kim M.-J., Chun B.-S. (2017). Effects of process parameters on EPA and DHA concentrate production from Atlantic salmon by-product oil: Optimization and characterization. Korean J. Chem. Eng..

[60] Koutsoukos S., Tsiaka T., Tzani A., Zoumpoulakis P., Detsi A. (2019). Choline chloride and tartaric acid, a Natural Deep Eutectic Solvent for the efficient extraction of phenolic and carotenoid compounds. J. Clean. Prod..

[61] da Silva Bambirra Alves F.E., Carpiné D., Teixeira G.L., Goedert A.C., de Paula Scheer A., Ribani R.H. (2021). Valorization of an Abundant Slaughterhouse By-Product as a Source of Highly Technofunctional and Antioxidant Protein Hydrolysates. Waste Biomass Valorization.

[62] Baş D., Boyacı İ.H. (2007). Modeling and optimization I: Usability of response surface methodology. J. Food Eng..

[63] Paul T., Halder S.K., Das A., Ghosh K., Mandal A., Payra P., Barman P., Das Mohapatra P.K., Pati B.R., Mondal K.C. (2015). Production of chitin and bioactive materials from Black tiger shrimp (*Penaeus monodon*) shell waste by the treatment of bacterial protease cocktail. 3 Biotech.

[64] Box G.E., Hunter J.S., Hunter W.G. (2005). Statistics for experimenters. Wiley Series in Probability and Statistics.

[65] Mousavi L., Tamiji Z., Khoshayand M.R. (2018). Applications and opportunities of experimental design for the dispersive liquid–liquid microextraction method—A review. Talanta.

[66] Ghorbannezhad P., Bay A., Yolmeh M., Yadollahi R., Moghadam J.Y. (2016). Optimization of coagulation–flocculation process for medium density fiberboard (MDF) wastewater through response surface methodology. Desalin. Water Treat..

[67] Sindhu S., Sherief P. (2011). Extraction, characterization, antioxidant and anti-inflammatory properties of carotenoids from the shell waste of arabian red shrimp *Aristeus alcocki*, ramadan 1938. Open Conf. Proc. J..

[68] da Silva Bernardo B., Kopplin B.W., Daroit D.J. (2023). Bioconversion of Fish Scales and Feather Wastes by *Bacillus* sp. CL18 to Obtain Protease and Bioactive Hydrolysates. Waste Biomass Valorization.

[69] Martí-Quijal F.J., Tornos A., Príncep A., Luz C., Meca G., Tedeschi P., Ruiz M.-J., Barba F.J. (2020). Impact of Fermentation on the Recovery of Antioxidant Bioactive Compounds from Sea Bass Byproducts. Antioxidants.

[70] Lee H.-J., Roy V.C., Ho T.C., Park J.-S., Jeong Y.-R., Lee S.-C., Kim S.-Y., Chun B.-S. (2021). Amino Acid Profiles and Biopotentiality of Hydrolysates Obtained from Comb Penshell (*Atrina pectinata*) Viscera Using Subcritical Water Hydrolysis. Mar. Drugs.

[71] Pal K., Rakshit S., Mondal K.C., Halder S.K. (2021). Microbial decomposition of crustacean shell for production of bioactive metabolites and study of its fertilizing potential. Environ. Sci. Pollut. Res..

[72] Mechri S., Sellem I., Bouacem K., Jabeur F., Laribi-Habchi H., Mellouli L., Hacène H., Bouanane-Darenfed A., Jaouadi B. (2020). A biological clean processing approach for the valorization of speckled shrimp *Metapenaeus monoceros* by-product as a source of bioactive compounds. Environ. Sci. Pollut. Res..

[73] Hamdi M., Hammami A., Hajji S., Jridi M., Nasri M., Nasri R. (2017). Chitin extraction from blue crab (*Portunus segnis*) and shrimp (*Penaeus kerathurus*) shells using digestive alkaline proteases from P. segnis viscera. Int. J. Biol. Macromol..

[74] Messina C.M., Manuguerra S., Arena R., Renda G., Ficano G., Randazzo M., Fricano S., Sadok S., Santulli A. (2021). In Vitro Bioactivity of Astaxanthin and Peptides from Hydrolisates of Shrimp (*Parapenaeus longirostris*) By-Products: From the Extraction Process to Biological Effect Evaluation, as Pilot Actions for the Strategy “From Waste to Profit”. Mar. Drugs.

[75] Messina C.M., Manuguerra S., Renda G., Santulli A. (2019). Biotechnological applications for the sustainable use of marine by-products: In vitro antioxidant and pro-apoptotic effects of astaxanthin extracted with supercritical CO_2_ from parapeneus longirostris. Mar. Biotechnol..

[76] Sedaghat F., Yousefzadi M., Toiserkani H., Najafipour S. (2016). Chitin from *Penaeus merguiensis* via microbial fermentation processing and antioxidant activity. Int. J. Biol. Macromol..

[77] Zhang H., Fang J., Deng Y., Zhao Y. (2014). Optimized production of *Serratia marcescens* B742 mutants for preparing chitin from shrimp shells powders. Int. J. Biol. Macromol..

[78] Wang M., Zhou J., Collado M.C., Barba F.J. (2021). Accelerated Solvent Extraction and Pulsed Electric Fields for Valorization of Rainbow Trout (*Oncorhynchus mykiss*) and Sole (*Dover sole*) By-Products: Protein Content, Molecular Weight Distribution and Antioxidant Potential of the Extracts. Mar. Drugs.

[79] Hamdi M., Nasri R., Dridi N., Li S., Nasri M. (2020). Development of novel high-selective extraction approach of carotenoproteins from blue crab (*Portunus segnis*) shells, contribution to the qualitative analysis of bioactive compounds by HR-ESI-MS. Food Chem..

[80] Lee S.-C., Nkurunziza D., Kim S.-Y., Surendhiran D., Singh A.A., Chun B.-S. (2022). Supercritical carbon dioxide extraction of squalene rich cod liver oil: Optimization, characterization and functional properties. J. Supercrit. Fluids.

[81] Islam N., Hoque M., Taharat S.F. (2023). Recent advances in extraction of chitin and chitosan. World J. Microbiol. Biotechnol..

[82] Ibram A., Ionescu A.-M., Cadar E. (2019). Comparison of extraction methods of chitin and chitosan from different sources. Eur. J. Nat. Sci. Med..

[83] Siddik M.A., Howieson J., Fotedar R., Partridge G.J. (2021). Enzymatic fish protein hydrolysates in finfish aquaculture: A review. Rev. Aquac..

[84] Aspevik T., Oterhals Å., Rønning S.B., Altintzoglou T., Wubshet S.G., Gildberg A., Afseth N.K., Whitaker R.D., Lindberg D. (2018). Valorization of proteins from co-and by-products from the fish and meat industry. Chemistry and Chemical Technologies in Waste Valorization.

[85] Mathew G.M., Huang C.C., Sindhu R., Binod P., Pandey A. (2022). Enzymes in seafood processing. Value-Addition in Food Products and Processing through Enzyme Technology.

[86] Halim N., Yusof H., Sarbon N. (2016). Functional and bioactive properties of fish protein hydolysates and peptides: A comprehensive review. Trends Food Sci. Technol..

[87] Dinakarkumar Y., Krishnamoorthy S., Margavelu G., Ramakrishnan G., Chandran M. (2022). Production and characterization of fish protein hydrolysate: Effective utilization of trawl by-catch. Food Chem. Adv..

[88] Gao R., Yu Q., Shen Y., Chu Q., Chen G., Fen S., Yang M., Yuan L., McClements D.J., Sun Q. (2021). Production, bioactive properties, and potential applications of fish protein hydrolysates: Developments and challenges. Trends Food Sci. Technol..

[89] Vázquez J.A., Meduíña A., Durán A.I., Nogueira M., Fernández-Compás A., Pérez-Martín R.I., Rodríguez-Amado I. (2019). Production of Valuable Compounds and Bioactive Metabolites from By-Products of Fish Discards Using Chemical Processing, Enzymatic Hydrolysis, and Bacterial Fermentation. Mar. Drugs.

[90] Muzaddadi A.U., Devatkal S., Oberoi H.S., Dhillon G.S., Kaur S. (2016). Chapter 9—Seafood Enzymes and Their Application in Food Processing. Agro-Industrial Wastes as Feedstock for Enzyme Production.

[91] Saranya R., Jayapriya J. (2018). Purification, characterization, molecular modeling and docking study of fish waste protease. Int. J. Biol. Macromol..

[92] Murthy L., Phadke G., Unnikrishnan P., Annamalai J., Joshy C., Zynudheen A., Ravishankar C. (2018). Valorization of fish viscera for crude proteases production and its use in bioactive protein hydrolysate preparation. Waste Biomass Valorization.

[93] Subramanian K., Sadaiappan B., Aruni W., Kumarappan A., Thirunavukarasu R., Srinivasan G.P., Bharathi S., Nainangu P., Renuga P.S., Elamaran A. (2020). Bioconversion of chitin and concomitant production of chitinase and N-acetylglucosamine by novel Achromobacter xylosoxidans isolated from shrimp waste disposal area. Sci. Rep..

[94] Affes S., Aranaz I., Hamdi M., Acosta N., Ghorbel-Bellaaj O., Heras Á., Nasri M., Maalej H. (2019). Preparation of a crude chitosanase from blue crab viscera as well as its application in the production of biologically active chito-oligosaccharides from shrimp shells chitosan. Int. J. Biol. Macromol..

[95] Doan C.T., Tran T.N., Nguyen V.B., Tran T.D., Nguyen A.D., Wang S.-L. (2020). Bioprocessing of squid pens waste into chitosanase by *Paenibacillus* sp. *TKU*047 and its application in low-molecular weight chitosan oligosaccharides production. Polymers.

[96] Silva A.K.N.d., Rodrigues B.D., Silva L.H.M.d., Rodrigues A.M.d.C. (2018). Drying and extraction of astaxanthin from pink shrimp waste (*Farfantepenaeus subtilis*): The applicability of spouted beds. Food Sci. Technol..

[97] Liu Z., Liu Q., Zhang D., Wei S., Sun Q., Xia Q., Shi W., Ji H., Liu S. (2021). Comparison of the proximate composition and nutritional profile of byproducts and edible parts of five species of shrimp. Foods.

[98] Hu J., Lu W., Lv M., Wang Y., Ding R., Wang L. (2019). Extraction and purification of astaxanthin from shrimp shells and the effects of different treatments on its content. Rev. Bras. Farmacogn..

[99] Li J., Sun W., Ramaswamy H.S., Yu Y., Zhu S., Wang J., Li H. (2017). High pressure extraction of astaxanthin from shrimp waste (*Penaeus Vannamei* Boone): Effect on yield and antioxidant activity. J. Food Process Eng..

[100] Shazana A.R., Masturah M., Badlishah S.B., Rashidi O., Russly A. (2016). Optimisation of supercritical fluid extraction of astaxanthin from Penaeus monodon waste using ethanol-modified carbon dioxide. J. Eng. Sci. Technol..

[101] Sánchez-Camargo A.P., Meireles M.Â.A., Ferreira A.L., Saito E., Cabral F.A. (2012). Extraction of ω-3 fatty acids and astaxanthin from Brazilian redspotted shrimp waste using supercritical CO_2_+ ethanol mixtures. J. Supercrit. Fluids.

[102] Cabanillas-Bojórquez L.A., Gutiérrez-Grijalva E.P., González-Aguilar G.A., López-Martinez L.X., Castillo-López R.I., Bastidas-Bastidas P.d.J., Heredia J.B. (2021). Valorization of fermented shrimp waste with supercritical CO_2_ conditions: Extraction of astaxanthin and effect of simulated gastrointestinal digestion on its antioxidant capacity. Molecules.

[103] Wang W., Liu M., Fawzy S. (2021). Effects of dietary Phaffia rhodozyma astaxanthin on growth performance, carotenoid analysis, biochemical and immune-physiological parameters, intestinal microbiota, and disease resistance in Penaeus monodon. Front. Microbiol..

[104] Gulzar S., Benjakul S. (2020). Impact of pulsed electric field pretreatment on yield and quality of lipid extracted from cephalothorax of Pacific white shrimp (*Litopenaeus vannamei*) by ultrasound-assisted process. Int. J. Food Sci. Technol..

[105] Poojary M.M., Barba F.J., Aliakbarian B., Donsì F., Pataro G., Dias D.A., Juliano P. (2016). Innovative alternative technologies to extract carotenoids from microalgae and seaweeds. Mar. Drugs.

[106] Costa D.d.S.V., Bragagnolo N. (2017). Development and validation of a novel microwave assisted extraction method for fish lipids. Eur. J. Lipid Sci. Technol..

[107] Barba F.J., Grimi N., Vorobiev E. (2015). New approaches for the use of non-conventional cell disruption technologies to extract potential food additives and nutraceuticals from microalgae. Food Eng. Rev..

[108] Schuur B., Brouwer T., Smink D., Sprakel L.M. (2019). Green solvents for sustainable separation processes. Curr. Opin. Green Sustain. Chem..

[109] Carpentieri S., Soltanipour F., Ferrari G., Pataro G., Donsì F. (2021). Emerging Green Techniques for the Extraction of Antioxidants from Agri-Food By-Products as Promising Ingredients for the Food Industry. Antioxidants.

[110] Awad A.M., Kumar P., Ismail-Fitry M.R., Jusoh S., Ab Aziz M.F., Sazili A.Q. (2021). Green Extraction of Bioactive Compounds from Plant Biomass and Their Application in Meat as Natural Antioxidant. Antioxidants.

[111] Saravana P.S., Ho T.C., Chae S.-J., Cho Y.-J., Park J.-S., Lee H.-J., Chun B.-S. (2018). Deep eutectic solvent-based extraction and fabrication of chitin films from crustacean waste. Carbohydr. Polym..

[112] El-Bialy H.A.A., Abd El-Khalek H.H. (2020). A comparative study on astaxanthin recovery from shrimp wastes using lactic fermentation and green solvents: An applied model on minced Tilapia. J. Radiat. Res. Appl. Sci..

[113] Vernes L., Abert-Vian M., El Maâtaoui M., Tao Y., Bornard I., Chemat F. (2019). Application of ultrasound for green extraction of proteins from spirulina. Mechanism, optimization, modeling, and industrial prospects. Ultrason. Sonochem..

[114] Fang Y., Gu S., Liu S., Zhang J., Ding Y., Liu J. (2018). Extraction of oil from high-moisture tuna liver by subcritical dimethyl ether: Feasibility and optimization by the response surface method. RSC Adv..

[115] Dutta S., Priyadarshini S., Moses J.A., Anandharamakrishnan C. (2021). Supercritical Fluid and Ultrasound-assisted Green Extraction Technologies for Catechin Recovery. ChemBioEng Rev..

